# Transverse‐Longitudinal Manipulation of High‐Order Bessel Vector Vortex Beams via Spin‐Multiplexed Metasurfaces

**DOI:** 10.1002/nap2.70364

**Published:** 2026-11-14

**Authors:** Lingling Xu, XueYing Wu, Mingli Sun, Xiaogang Wang, Xinning Yu, Tingting Sun, Bijun Xu

**Affiliations:** ^1^ Department of Physics Zhejiang University of Science and Technology Hangzhou China

**Keywords:** bessel beam, longitudinal evolution, metasurfaces, polarization order, spin multiplexing, vector beams

## Abstract

Vector beams, structured optical fields with spatially variant polarization distributions, play vital roles in optical manipulation, super‐resolution imaging, optical communication, and beyond. Conventional generation of longitudinally evolving vector beams relies on bulky optical components with poor system integration and limited modulation freedom. This work presents a spin‐multiplexed metasurface approach to generate high‐order Bessel vector vortex beams, enabling simultaneous transverse and longitudinal polarization control. For a fixed topological‐charge difference, the relative difference in the main‐ring radius of high‐order Bessel beams decreases significantly as the topological charge increases. Independent design of polarization orders for multiple angular sectors is achieved via phase modulation of orthogonal circularly polarized components and segmented encoding strategy. The phase‐mapping relationship of Bessel axicons enables longitudinally segmented evolution of polarization orders and net orbital angular momentum (OAM). The simulation results preliminarily indicate that the proposed design is promising for combined transverse–longitudinal modulation, same‐scale superposition of high‐order topological charges, independent design of polarization orders in multiple angular sectors, and segmented longitudinal evolution of the polarization order *P*(*z*) and the net orbital angular momentum (OAM) *L*(*z*). This scheme may provide a potential pathway for structured light field manipulation, on‐chip integrated photonics, and high‐capacity structured optical communication.

## Graphical abstract

A spin‐multiplexed metasurface decomposes x‐linearly polarized incidence into LCP and RCP components with independently encoded phases. The high‐order Bessel strategy enables efficient spatial overlap of the two spin components within the same transverse scale, thereby realizing a transversely nonuniform polarization‐order distribution and piecewise‐continuous evolution of *P*(*z*) and *L*(*z*) along the propagation direction.

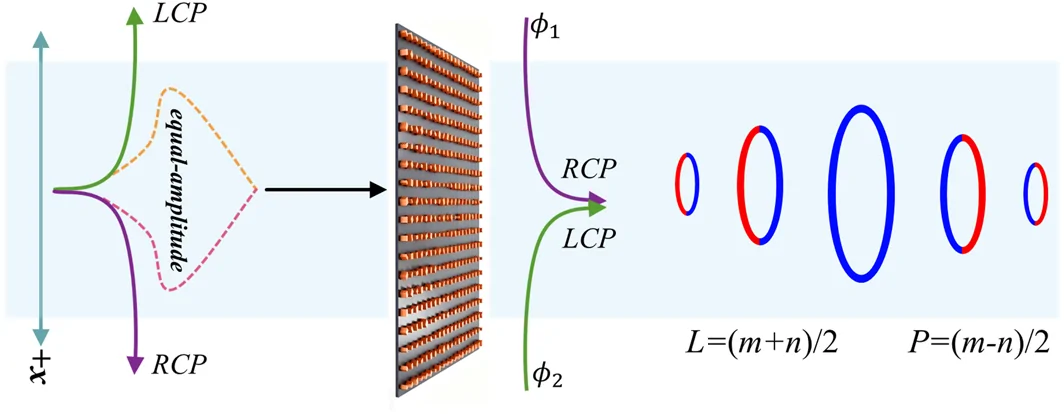

## 1 Introduction

Vector beams have attracted considerable attention owing to their unique application potentials in optical trapping [1], high‐resolution imaging [2], and optical communications [3]. Beyond static transverse polarization distributions, vector optical fields that evolve longitudinally along the propagation direction offer an additional degree of freedom for beam shaping [4], laser processing [5], and information multiplexing [6]. However, conventional generation methods rely on cascaded optical components such as spatial light modulators and q‐plates, which suffer from inherent drawbacks including bulky system volume and complex optical alignment, severely limiting the development of integrated photonic devices. In contrast, metasurfaces, as planar optical components capable of flexibly manipulating electromagnetic fields at the subwavelength scale, provide a viable route to compact and chip‐integrable vector beams generation [7].

Recent studies reveal that the research on vector optical fields generated by metasurfaces is evolving from static transverse manipulation to multi‐dimensional dynamic manipulation [4, 8, 9, 10]. Currently, the generation technology of two‐dimensional vector beams has matured considerably. Spatially variant polarization patterns such as radial, azimuthal and vortex polarization can be realized at the subwavelength scale via Jones matrix modulation strategies including geometric phase and propagation phase [11, 12]. For instance, Cheng et al. achieved efficient eight‐channel decoupled modulation using a bilayer triatomic metasurface [13]. In addition, Wang et al. proposed an inhomogeneous full‐matrix Jones matrix strategy for arbitrary vectorial optical fields [14]. Nevertheless, the aforementioned methods are still limitedto static transverse polarization distribution, with the polarization state invariant along the beam propagation direction or only designed on a target plane.

To overcome this limitation, researchers have extended polarization manipulation to the longitudinal degree of freedom. Previous studies have adopted long‐depth‐of‐focus or multi‐focal‐plane designs, in which two vortex beams with different focal lengths and different topological‐charge combinations are integrated into orthogonal circular polarization channels. The focal depths of the two channels partially overlap, and their intensity ratio and relative phase vary with propagation distance, so that the dominant component switches and the polarization state or topological charge evolves continuously along the propagation direction; however, this longitudinal behavior is governed by the coupled focusing process, making it difficult to independently set the target state at a designated propagation plane [8, 15, 16]. To realize propagation‐distance‐dependent polarization manipulation, researchers have further extended metasurface designs to Bessel‐like beam systems. Within this framework, Yang et al. exploited the non‐diffracting property of Bessel beams and introduced wave vector difference between orthogonal circular polarization components to generate phase differences that accumulate linearly with propagation distance, thereby realizing continuous rotation of the polarization angle along propagation, periodically switching between *x*‐ and *y*‐linear polarizations [4]. However, the polarization state in each transverse plane remains uniformly linear, lacking spatially variant polarization distribution. Zhou et al. adopted spin‐multiplexed metasurfaces and assigned distinct numerical apertures to the LCP and RCP components, achieving continuous longitudinal evolution of the polarization state from radial to azimuthal polarization in the visible band [9]. However, this evolution exhibits periodic switching rather than an arbitrarily customizable longitudinal evolution pattern. Nevertheless, most of these schemes still rely on globally cylindrically symmetric polarization modes or a single polarization order.

Meanwhile, the broader structured‐light literature has developed several approaches to decouple or engineer OAM from the radial intensity distribution [17, 18, 19]. These works mainly focus on perfect OAM, high‐purity OAM, or intensity‐pattern engineering on a single transverse plane. In contrast, this work does not aim to generate perfect OAM or arbitrary intensity patterns. Instead, it adopts a high‐order Bessel strategy and exploits the fact that, for a fixed topological‐charge difference, the relative difference in the main‐ring radius of high‐order Bessel beams decreases significantly as the topological charge increases, enabling two spin components carrying different topological charges to achieve highly efficient spatial overlap within the same transverse scale, thereby avoiding the mode‐separation issue caused by the main‐ring radius mismatch in low‐order Bessel beams.

Based on this overlap, this work further realizes a nonuniform transverse polarization‐order distribution and segment‐wise continuous evolution of *P*(*z*) and *L*(*z*) along the propagation direction through radial zoning and azimuthal sector encoding. Accordingly, we report a transmissive high‐order Bessel vector vortex beam (BVVB) enabled by form‐birefringent metasurfaces. Unlike previous strategies restricted to a uniform global polarization order or static transverse polarization grafting [20], this beam supports both features simultaneously during propagation, thereby surpassing cylindrically symmetric modes with a single global polarization order (Table 1).

**TABLE 1:** Comparison of polarization evolution scheme, design complexity, and efficiency between this work and representative existing works.

Reference	Polarization evolution scheme	Design complexity	Efficiency
This work	Piecewise‐continuous longitudinal evolution with independent design within each propagation interval	Single‐layer dielectric metasurface; spin multiplexing; segmented annular zones	T_MS_ = 0.702; average cross‐polarization efficiency $\eta_{\text{cross}}$ = 0.929; target OAM mode dominated; average 87.79%
Ref. [4]	Continuous rotation of polarization angle; uniform linear polarization in each transverse plane	Birefringent dielectric metasurface; visible band	Not quantitatively reported
Ref. [8]	Continuous; ellipticity and azimuth variation	All‐silicon metasurface; long depth‐of‐focus design; LCP/RCP focal depths of 4–6.5 mm and 6–8.5 mm	100% cross‐polarization conversion rate (PCR)
Ref. [9]	Continuous; radial‐to‐azimuthal periodic switching	Single‐layer dielectric metasurface; continuous axicon phase (NA_1_ = 0.12; NA_2_ = 0.09)	Working efficiency ≈ 43.2% and 43.1% (single VOF); 41.5% (dual VOF)
Ref. [10]	Continuous rotation of polarization angle; uniform linear polarization in each transverse plane	Surface‐wave excitation; PB meta‐atoms; microwave band	PCR = 0.11 (12 GHz, intentionally low)
Ref. [15]	Continuous longitudinal evolution; topological charge and polarization state vary with propagation distance	All‐silicon metasurface; long depth‐of‐focus design; propagation phase and geometric phase	Not quantitatively reported
Ref. [16]	Continuous longitudinal evolution; azimuthal to radial and azimuthal to second‐order radial	3D‐printed alumina metasurface; propagation phase and geometric phase; long depth‐of‐focus design	Focusing efficiency ≈ 26.70% (device I) and 29.63% (device II) experimentally; simulation ≈ 37.63% and 38.51%
Ref. [20]	No longitudinal evolution; transversely varying polarization order	Plasmonic metasurface; grafted perfect vortex beams; dynamic tuning via a rotating half‐wave plate	Relatively low conversion efficiency (plasmonic Au nanorods)

*Note:* The total transmittance *T*_MS_ = 0.702 is the full‐aperture transmittance normalized to the incident power. The average cross‐polarization efficiency is the average over the 12 selected nanopillar unit cells and the cross‐polarization efficiency is defined as $\eta_{\text{cross}}^{\left(i\right)}=\text{PC}R^{\left(i\right)}\times T_{\text{avg}}^{\left(i\right)}$. where $\text{PC}R^{\left(i\right)}=\sin^{2}\left(\Delta \phi^{\left(i\right)}/2\right)$ is the polarization conversion rate, and $T_{\text{avg}}^{\left(i\right)}=\left(T_{x}^{\left(i\right)}+T_{y}^{\left(i\right)}\right)/2$ is the average transmittance of the unit cell. The PCR is determined by the phase difference between the *x* and *y* principal axes of the nanopillar, while the transmittance determines how much incident energy is transmitted. Their product gives the fraction of incident light converted into the cross‐polarized component. Target OAM mode purity = sum of the target‐mode components in the normalized OAM spectrum.

## 2 Theory

A Bessel vector vortex beam is a type of structured optical field characterized by spatially variant polarization distribution, a helical wavefront, and nondiffracting propagation properties [21, 22]. Because its electric field distribution is described by Bessel functions, the beam maintains a constant transverse spot size during propagation, thereby overcoming the diffraction divergence inherent in ordinary vector vortex beams. Under the paraxial approximation, a Bessel vector vortex beam can be expressed as the coherent superposition of two orthogonally circularly polarized Bessel vortex components [23]. Assuming that the RCP component and the LCP component carry topological charges *m* and *n*, respectively, and both share the same transverse wavenumber $k_{\rho }$, the total electric field can be written as:

(1) $$E\left(\rho ,\phi ,z\right)\;\propto \;J_{m}\left(k_{\rho }\rho \right)e^{im\phi }|R\rangle +J_{n}\left(k_{\rho }\rho \right)e^{in\phi }|L\rangle$$

where, $J_{m}$ and $J_{n}$ are Bessel functions of the first kind, |R〉 and |L〉 are the circular polarization basis vectors, and ϕ is the azimuthal angle. To separate the overall helical phase and the polarization distribution [9], a common phase factor $e^{i\left(m+n\right)\phi /2}$ is extracted:

(2) $$E\;\propto \;e^{iL\phi }\left(e^{iP\phi }|R\rangle +e^{-iP\phi }|L\rangle \right)$$

where,

(3) $$L=\frac{m+n}{2},P=\frac{m-n}{2}$$

L denotes the net OAM of the beam, which shapes its helical wavefront; each photon carries an OAM value of Lℏ. P is referred to as the polarization order, which governs the variation of the polarization state with the azimuthal angle ϕ.

When using metasurfaces to generate BVVB, the axicon phase is typically encoded directly into the phase response. In cylindrical coordinates (ρ,ϕ,z), the axicon phase can be expressed as:

(4) $$\phi_{\text{axicon}}=-k\cdot \text{NA}\cdot \rho$$

where, k=2π/λ is the wavenumber in free space, λ is the wavelength, and NA is the numerical aperture [22, 24]. According to generalized Snell's law, the Bessel beam generated by an axicon satisfies a linear mapping between the propagation distance *z* and the radial coordinate ρ:

(5) ρ=z·tanθ

where, θ is the cone angle, and sinθ=NA.

For high‐order Bessel functions ($J_{l}\left(x\right)$ with l≫1), the first positive zero $j_{l,1}$ has a uniform asymptotic expansion:

(6) $$x_{l,1}∼l+a_{1}{\left(\frac{l}{2}\right)}^{1/3}+\frac{3}{10}{a_{1}}^{2}{\left(\frac{l}{2}\right)}^{-1/3}+\text{⋯}$$

where, $a_{1}\approx 2.33811$ is the first zero of the Airy function Ai(x) [25]. This expansion indicates that the leading term l causes the zero to increase linearly with the order, while the correction term $a_{1}l^{1/3}$ grows much more slowly than l. Consequently, as the order l increases, the growth rate of the zero $x_{l,1}$ continuously decreases. The rate of change of $x_{l,1}$ with respect to l is given by:

(7) $$\frac{dx_{l,1}}{dl}=\frac{2}{\pi x_{l,1}{\left[Y_{l}\left(x_{l,1}\right)\right]}^{2}}$$

where, $Y_{l}\left(x\right)$ is the Bessel function of the second kind [26]. In the high‐order regime (l≫1), both $x_{l,1}$ and $Y_{l}\left(x_{l,1}\right)$ are large in magnitude, resulting in a significant increase in the denominator of the derivative expression, and thus the value of $dx_{l,1}/dl$ becomes extremely small.

According to the relation $\rho =\frac{x_{l,1}\lambda }{2\pi NA}$, the radius ρ of the main ring of the Bessel beam is directly determined by $x_{l,1}$ for fixed wavelength λ and numerical aperture NA. For a fixed topological‐charge difference (e.g., Δl=2), the absolute radius difference Δρ=ρ(l+1)−ρ(l−1) approaches a nonzero constant as l increases, about 0.34 μm under the present parameters. The relative radius difference Δρ/ρ(l) decreases significantly with increasing l and tends to zero. Therefore, for a fixed topological‐charge difference, the relative difference in the main‐ring radius of high‐order Bessel beams decreases significantly as the topological charge increases, which facilitates same‐transverse‐scale superposition of different topological charges. The comparison between the asymptotic formula and the numerical solution, the quantitative comparison between low and high orders, and the full‐wave FDTD verification are provided in Supporting Information S1: Note S1.

To realize combined transverse and longitudinal modulation, we adopt a joint encoding strategy combining radial rings and angular sectors. The radial domain is divided into N annular zones, where the radial range of the *i*th annular zone is $\left(\rho_{i-1},\rho_{i}\right)\left(\rho_{0}=0,\rho_{N}=R\right)$. The net OAM $L_{i}$ is independently assigned to each annular zone. Meanwhile, within each annular zone, the transverse plane is equally divided into M sectors along the azimuthal direction, and the azimuthal range of the *k*th sector is $\left[\frac{2\pi }{M}\left(k-1\right),\frac{2\pi }{M}k\right]$. Equation (3) can then be specifically written as:

(8) m(ρ,ϕ)=L(ρ)+P(ρ,ϕ),n(ρ,ϕ)=L(ρ)−P(ρ,ϕ)

where, L(ρ) and P(ρ,ϕ) are a radially piecewise‐constant function and a radially–azimuthally jointly piecewise‐constant function, respectively. Their explicit definitions and the description of the indicator function are provided in Supporting Information S1: Note S2.

To eliminate the phase discontinuity at the sector boundaries [27], phase‐offset compensation terms $\delta^{\left(m\right)}$ and $\delta^{\left(n\right)}$ are introduced. These compensation terms are entirely determined by the distribution of the polarization order P within each sector, and the detailed derivation is provided in Supporting Information S1: Note S3.

In this design, M=2 is taken, that is, the cross‐section is divided into two sectors, A and B. The polarization orders of the two sectors are usually set as opposite numbers, and their absolute values are both set to 1.

(9) $$P_{i,B}=-P_{i,A},\left|P_{i,A}\right|=\left|P_{i,B}\right|=1$$

To achieve the aforementioned independent phase control, a spin‐multiplexed metasurface combining dynamic phase and Pancharatnam–Berry phase is employed. Specifically, consider a metasurface described by a Jones matrix J(x,y). Under the circular polarization basis |L〉, |R〉, to achieve spin decoupling, the metasurface should satisfy the following input–output relationship:

(10) $$J\left(x,y\right)|L\rangle =e^{i\phi_{1}\left(x,y\right)}|R\rangle ,J\left(x,y\right)|R\rangle =e^{i\phi_{2}\left(x,y\right)}|L\rangle$$

That is, LCP incidence is converted to RCP emission carrying a phase $\phi_{1}$, and vice versa. This condition ensures that the two orthogonal circular polarization components can independently acquire different phase distributions $\phi_{1}$ and $\phi_{2}$.

To realize the aforementioned Jones matrix, anisotropic nanopillars are typically employed as meta‐atoms. The dynamic phase is determined by the values of $\phi_{x}$ and $\phi_{y}$; the geometric phase is determined by the rotation angle θ , producing a phase modulation of ± 2θ for circularly polarized light. To achieve efficient cross‐polarization conversion [28], the half‐wave plate condition must be satisfied:

(11) $$\phi_{x}-\phi_{y}=\pi$$

In this case, the phases $\phi_{1}$ and $\phi_{2}$ acquired by the two orthogonal circular polarization components upon passing through the metasurface are related to the nanopillar dimensions by [29]

(12) $$\phi_{x}=\frac{\phi_{1}+\phi_{2}}{2},\phi_{y}=\frac{\phi_{1}+\phi_{2}}{2}-\pi ,\theta =\frac{\phi_{1}-\phi_{2}}{4}$$

We design two metasurfaces, denoted as $MS_{1}$ and $MS_{2}$. $MS_{1}$ adopts a set of phase pairs with NA=0.6, L varies symmetrically along the radial direction, taking the value sequence 60, 70, 80, 70, 60. The target phases $\phi_{1}$ and $\phi_{2}$ for the LCP and RCP components of $MS_{1}$ are constructed by superposing an axicon phase, a vortex phase, and a phase‐offset compensation term, expressed as:

(13) $$\begin{array}{l} \phi_{1}\left(\rho ,\phi \right)=-k\cdot \text{NA}\cdot \rho +m\left(\rho ,\phi \right)\cdot \phi +\delta^{\left(m\right)}\left(\rho ,\phi \right)\\ \phi_{2}\left(\rho ,\phi \right)=-k\cdot \text{NA}\cdot \rho +n\left(\rho ,\phi \right)\cdot \phi +\delta^{\left(n\right)}\left(\rho ,\phi \right) \end{array}$$

The axicon phase generates the Bessel beam and establishes the radial‐longitudinal mapping. The vortex phase carries orbital angular momentum. The phase design for $MS_{2}$ is provided in the Supporting Information S1: Note S4.

Figure 1a presents the schematic principle, illustrating how to generate high‐order Bessel vector vortex beams with nonuniform transverse polarization order distributions and segment‐wise continuous longitudinal evolution of both the polarization order and the net OAM. Figure 1b presents the designed phase distributions $\phi_{1}$ and $\phi_{2}$ for $MS_{1}$. Figure 1c presents the main‐ring radius difference Δρ between two Bessel beams with a topological charge difference of 2 as a function of L. This topological charge difference corresponds exactly to that between the LCP and RCP components when the polarization order is *P* = ± 1 (i.e., m−n=±2), thus reflecting the spatial overlap of the two spin components. As L increases, Δρ=ρ(L+1)−ρ(L−1) gradually stabilizes and converges to approximately 0.34 μm. The relative difference Δρ/ρ(L) decreases rapidly from 8.66% at L=20 to 3.09% at L=60, and further to 2.35% at L=80. For a fixed topological‐charge difference, the relative difference in the main‐ring radius of high‐order Bessel beams therefore decreases significantly as the topological charge increases, indicating that the radial misalignment between the two spin components becomes negligibly small compared with the ring radius itself and ensuring high‐quality vector beam formation upon their superposition. For > 60, the improvement in the relative difference slows down, with an average reduction of only about 0.7% points per increment of 20 in L.

**FIGURE 1:**
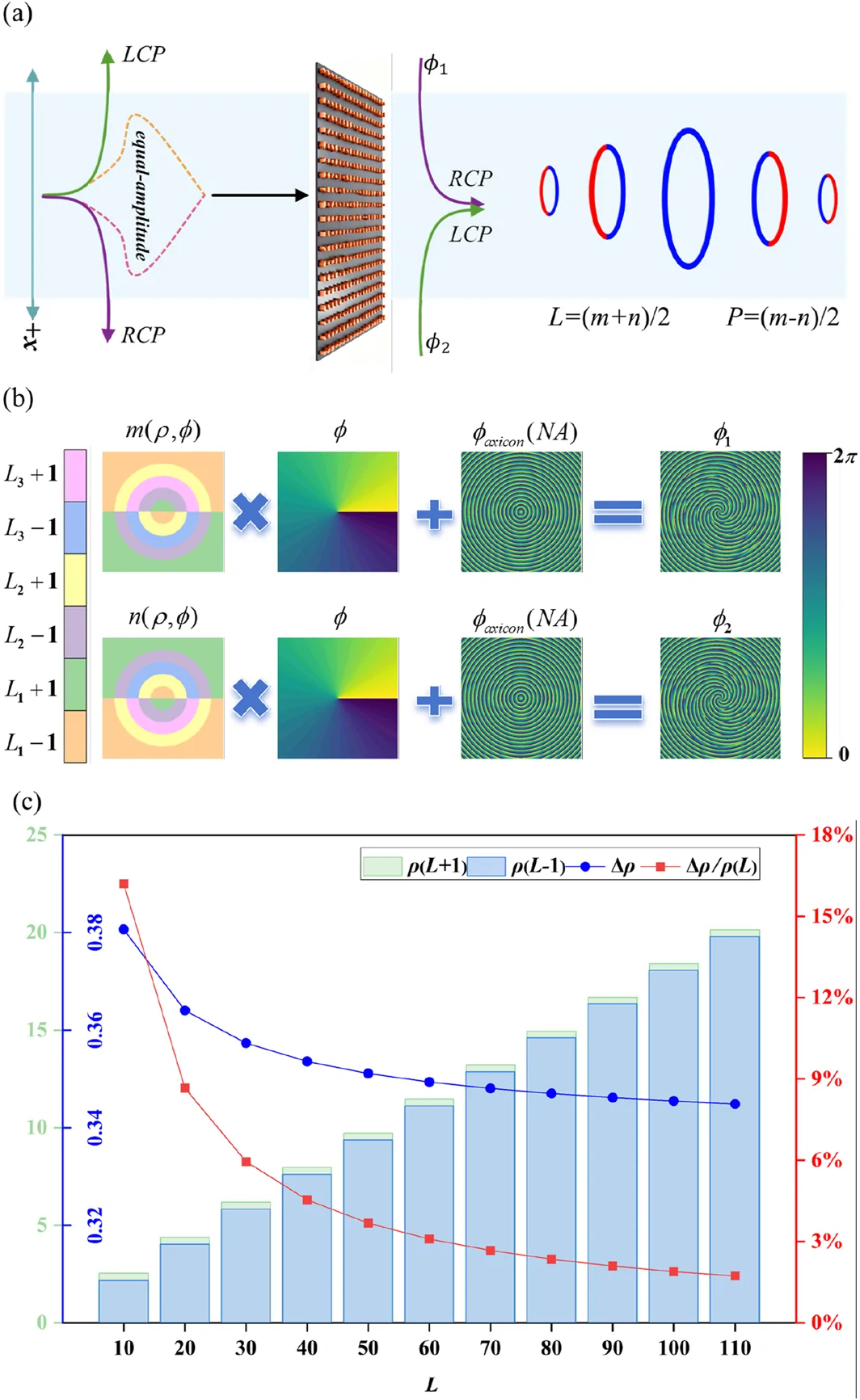
Generation principle of combined transverse and longitudinal modulation high‐order BVVBs via spin‐multiplexed metasurfaces. (a) Schematic illustration. The incident beam is x‐linearly polarized and equally decomposed into LCP and RCP components. The phases $\phi_{1}$ and $\phi_{2}$ correspond to the LCP‐to‐RCP and RCP‐to‐LCP conversion channels, carrying topological charges m and n, respectively. By independently designing $\phi_{1}$ and $\phi_{2}$ with radial‐zone and angular‐sector synergistic encoding (see (b)), the polarization order and net OAM are jointly tailored, where P=(m−n)/2 and L=(m+n)/2. Red/blue sectors denote P=−1 and P=+1, respectively. The single ring denotes the main lobe of the high order BVVBs, with its radius determined by the L sequence. (b) Designed phase distributions $\phi_{1}$ and $\phi_{2}$ for $MS_{1}$. The left colorbar indicates discrete m and n values. Column 1 shows the topological charge (m or n) distribution, column 2 the azimuthal angle φ, and column 3 the NA‐dependent axicon phase; the vortex phase is mφ or nφ. (c) Theoretical main‐ring radius difference Δρ=ρ(L+1)−ρ(L−1) between two Bessel beams with a topological charge difference of 2 as a function of L.

## 3 Simulation and Results Analysis

Both the unit‐cell and full‐metasurface optical responses were simulated using the finite‐difference time‐domain (FDTD) method. In the unit‐cell simulations, periodic boundary conditions were applied in the x and y directions, and perfectly matched layers were used along the propagation direction. In the full‐metasurface simulations, the array size was 511 × 511, and the aperture was 214 μm. The incident light was a normally incident x‐linearly polarized plane wave at 633 nm. Transmission and field distributions were recorded by power monitors and field monitors placed at the observation planes. The unit cell structure is shown in Figure 2a. The metasurface consists of $\text{Ti}O_{2}$ nanopillars on a $\text{Si}O_{2}$ substrate. The nanopillar height is fixed at H = 600 nm, and the lattice period is set to P = 420 nm, which is smaller than the operating wavelength λ = 633 nm. Figure 2b presents the simulated phase and transmittance for x‐ and y ‐polarized incidence at 633 nm, with white dots marking the 12 structural parameters selected for constructing the metasurface. Figure 2c shows the transmittance and phase delay characteristics of the 12 selected unit cells under x‐ and y ‐polarized incidence. These cells provide full 0–2π phase control and maintain a transmittance consistently above 0.7, meeting the requirements for high‐efficiency metasurface design [30]. The cross‐polarization efficiency $\eta_{\text{cross}}$, calculated by Jones matrix analysis, ranges from 73.7% to 99.7%, with an average of approximately 93.9%. Detailed unit‐cell data are provided in Supporting Information S1: Note S5. Figure 2d shows the complete design flow of the metasurface layout. From the target phase distributions $\phi_{1}$ and $\phi_{2}$ for the LCP and RCP components, the required phase delays along the two principal axes and the in‐plane rotation angle are determined, and the optimal nanopillar length and width are then selected from the pre‐established unit‐cell library.

**FIGURE 2:**
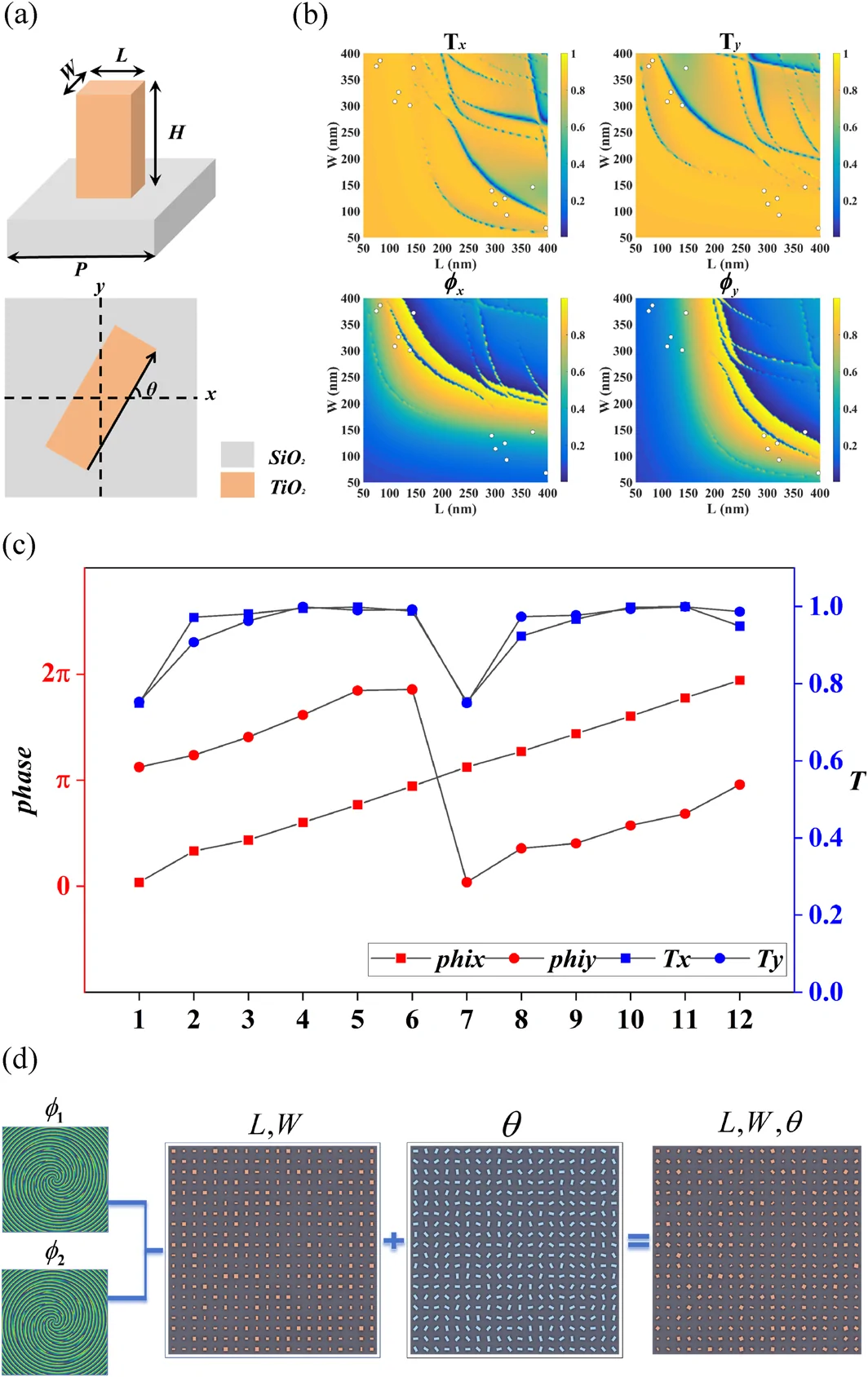
(a) Schematic diagram of the metasurface unit cell. (b) FDTD‐simulated phase and transmittance for x‐ and y‐polarized incidence (λ = 633 nm). The white dots mark the 12 structural parameters selected for constructing the metasurface. (c) Phase delay (in units of 2π) and normalized transmittance of the 12 selected unit cells under x‐ and y‐polarized incidence. (d) Design workflow of the metasurface layout.

The axicon phase establishes a linear mapping between the radial coordinate ρ and the longitudinal distance z. Therefore, the radial zoning determines the longitudinal sequence of the main‐ring radius. The angular sector encoding independently assigns the polarization order P within each sector, thereby controlling the azimuthal rotation direction of the bright and dark lobes. Because opposite polarization orders correspond to opposite signs of the geometric phase gradient, when the signs of P in the two sectors are opposite, the two sectors exhibit opposite rotation directions; when the signs of P in the two sectors are the same, the rotation directions are identical. Figure 3 presents the intensity distributions of the beam generated by $MS_{1}$ at five propagation distances for five linear polarization directions. When the polarization orders of the two sectors have opposite signs ($z_{1}$, $z_{2}$, $z_{4}$, and $z_{5}$ planes), the intensity patterns exhibit asymmetric spiral distributions, with the bright and dark lobes rotating in opposite directions (clockwise and counterclockwise) in the two sectors, confirming the asymmetric rotation characteristic. In contrast, when the polarization orders have the same sign ($z_{3}$ plane), the patterns exhibit symmetric distributions with consistent rotation directions. The number of bright lobes in each sector is given by 2|P|/M, which equals 1 in this design.

**FIGURE 3:**
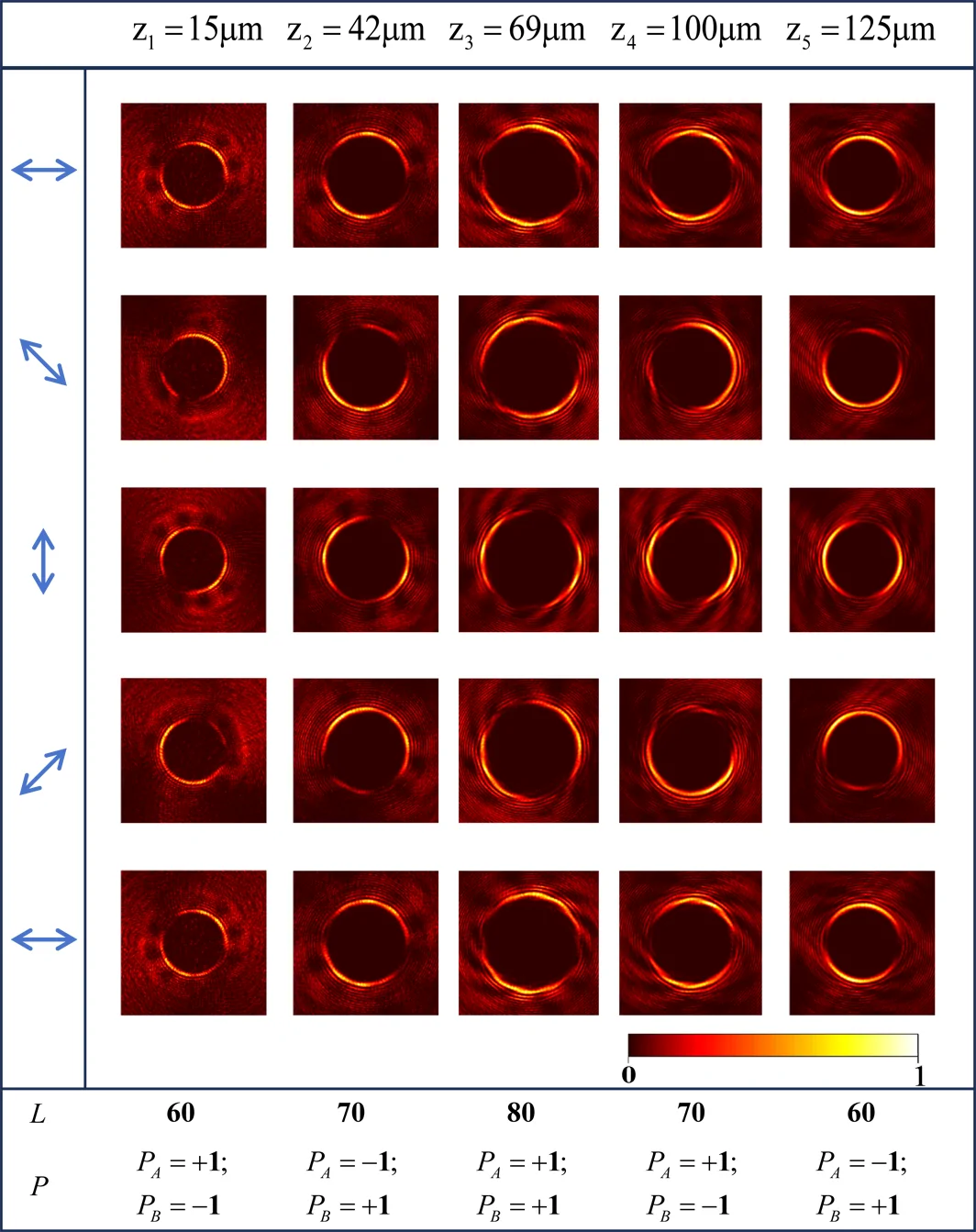
Presents the intensity distributions of the beam generated by $MS_{1}$ at five propagation distances, corresponding to linear polarization directions of 0°, 45°, 90°, 135°, and 180° (0° denotes *x*‐direction linear polarization, and 90° denotes *y*‐direction linear polarization). The designed net OAM and polarization order for each *z*‐plane are labeled in the figure, with blue arrows indicating the linear polarization directions. For each plane, the five polarization intensity images are normalized to the maximum intensity among the five polarization states, and all subfigures share the same color bar (0 → 1) representing relative intensity. All subfigures share the same coordinate range of ± 20 μm in both *x* and *y*.

Figure 4a presents a systematic analysis of the transverse–longitudinal transmission characteristics of the optical field. The y–z plane intensity distribution shows the longitudinal evolution of the beam. The transverse (x–y) intensity distributions of the LCP and RCP components at five propagation distances reveal a high degree of overlap in the main‐ring radial position, as confirmed by the radial intensity profiles along y = 0. The total intensity distributions show that the main‐ring radial position first increases and then decreases with propagation distance, consistent with the longitudinal evolution of the net OAM. At all observation planes, the intensity distributions exhibit smooth annular profiles without significant side lobes or background noise.

**FIGURE 4:**
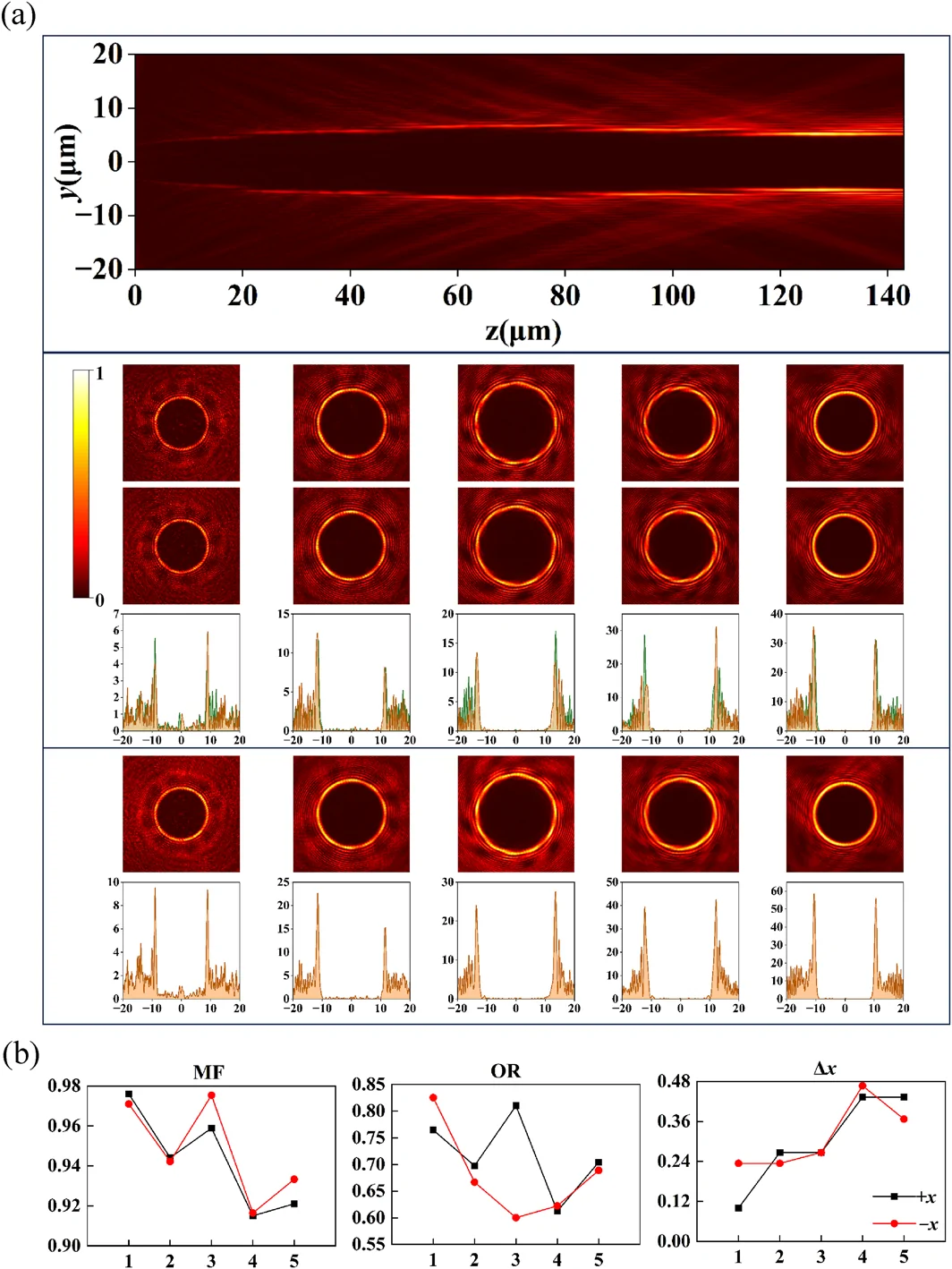
Longitudinal and transverse analysis of the optical field. (a) The upper panel shows the longitudinal intensity evolution in the *y*–*z* plane. The middle panels present the transverse (*x*–*y*) intensity distributions of the LCP and RCP components at five propagation distances, both normalized to the maximum of the two components within each plane, together with the corresponding radial intensity profiles along *y* = 0. The lower panels show the total intensity distributions, normalized to their own maximum intensity, with their radial profiles along *y* = 0. All subfigures share the same coordinate range of ± 20 μm in both *x* and *y*. (b) Statistical data of the matching factor (MF), overlap ratio (OR), and peak position difference (Δx) for the LCP and RCP components at different propagation distances.

To quantitatively evaluate the spatial overlap between the LCP and RCP components within the main‐ring region, three metrics are defined: the peak position difference Δx, the common overlap ratio OR, and the matching factor MF. The detailed definitions and calculation procedures of these metrics are provided in Supporting Information S1: Note S6. Figure 4b presents the MF, OR, and Δx for the LCP and RCP components on the x < 0 and x > 0 sides at different propagation distances. Across the non‐diffracting region, at five selected propagation distances, MF ranges from 0.910 to 0.975, OR varies between 0.62 and 0.83, and Δx lies between 0.11 and 0.48 μm. These results indicate that the LCP and RCP components maintain excellent spatial matching, with highly similar intensity profiles within the overlapping region.

To evaluate the transverse quality and longitudinal manipulation capability of the vector beam generated by the designed metasurface, horizontal intensity profiles are extracted at different propagation distances, and the symmetry, radial size, and full width at half maximum (FWHM) of the main ring are quantitatively analyzed.

The main‐ring radius is defined as the average of the absolute values of the left and right peak centers in the horizontal profile, that is, the radial coordinate at which the intensity reaches its maximum. The theoretical radius is determined by the first positive zero $j_{l,1}^{\prime }$ of the derivative of the *l*th order Bessel function ${J_{l}}^{\prime }\left(x\right)$, and is calculated as $\rho =j_{l,1}^{\prime }\lambda /\left(2\pi NA\right)$, where $j_{l,1}^{\prime }$ is obtained by numerically solving ${J_{l}}^{\prime }\left(x\right)=0$.

The FWHM is defined as the radial width at half the peak intensity. To quantitatively evaluate the circular symmetry of the transverse intensity distribution of the vector beam, the relative difference between the left and right FWHM values is introduced:

(14) $$\delta_{FWHM}=\frac{\left|\text{FWH}E_{L}-\text{FWH}E_{R}\right|}{\left(\text{FWH}E_{L}+\text{FWH}E_{R}\right)/2}\times 100\%$$

The closer this metric is to 0, the closer the beam is to a perfectly circularly symmetric annular intensity distribution.

Figure 5a presents the symmetry and radial‐size analysis of the main ring. The absolute values of the two peak centers are strictly equal at *z* = 42, 69, 95, and 125 μm, with deviations below 0.001 μm. At *z* = 15 μm, the two peak centers are located at −8.957 μm and +8.991 μm, with an absolute deviation of 0.034 μm and a relative deviation of only 0.38%, confirming the excellent radial symmetry of the total intensity distribution. The measured radius increases from 8.974 μm at *z* = 15–13.561 μm at *z* = 69 μm, and then decreases to 10.559 μm at *z* = 125 μm. This “first‐increase‐then‐decrease” trend is in excellent agreement with the designed *L* sequence.

**FIGURE 5:**
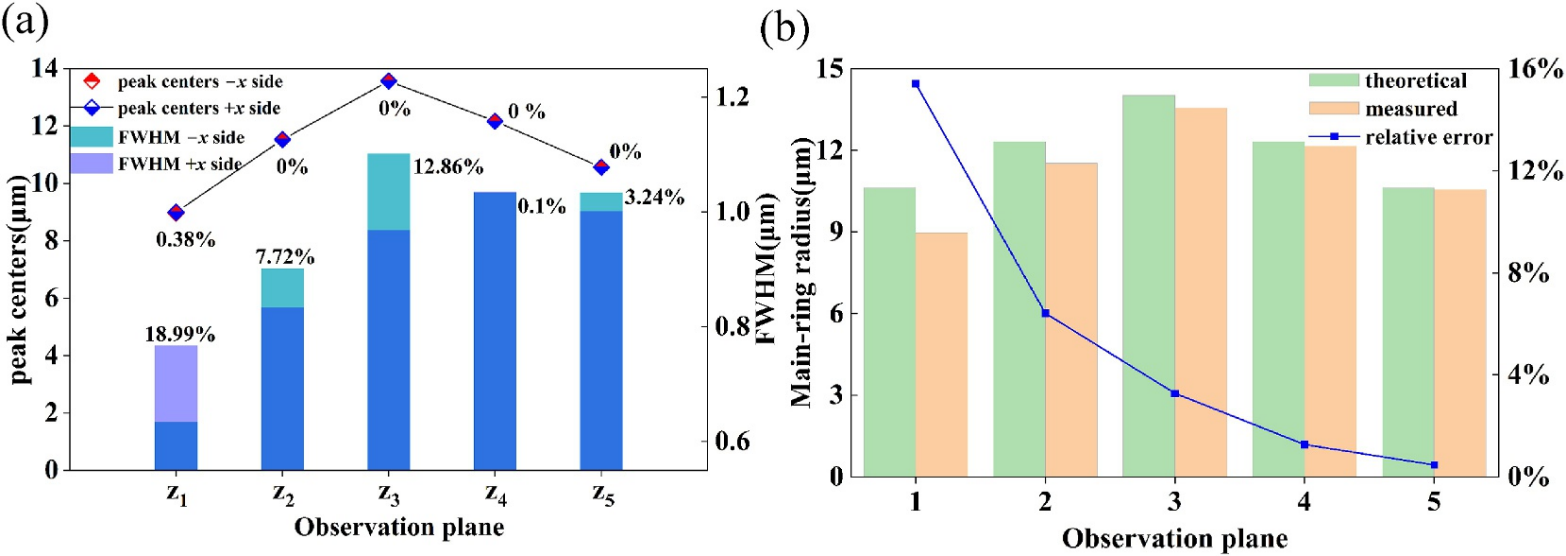
(a) Relative differences of the peak‐center positions and FWHM between the left and right sides of the main ring at five propagation distances ($z_{1}$–$z_{5}$). (b) Comparison of the theoretical main ring radii (green bars), measured radii (light orange bars), and radius relative errors (blue line with markers) at each observation plane.

At *z* = 15 μm, the relative difference in FWHM between the two sides reaches its maximum of 18.99% (0.634 μm on the *x* < 0 side and 0.767 μm on the *x* > 0 side), indicating slight elliptical astigmatism in the near‐field region. As the propagation distance increases, the asymmetry gradually diminishes, and the difference decreases rapidly: at *z* = 95 μm, the two side widths are almost identical (1.034 vs. 1.033 μm, with a difference of 0.1%), and at *z* = 125 μm, the difference further decreases to 3.24% (1.034 μm on the *x* < 0 side and 1.001 μm on the *x* > 0 side).

Figure 5b compares the theoretical radii (green bars), measured radii (light orange bars), and relative errors of the radius (blue line) of the Bessel beam main ring at five observation planes. The theoretical main‐ring radii corresponding to *L* = 60, 70, and 80 are 10.609 μm, 12.316 and 14.020 μm, respectively. At all observation planes, the measured radius are smaller than the theoretical values, with the relative error decreasing continuously with propagation distance from 15.41% to 0.47%. This deviation mainly originates from the NA‐related phase‐gradient error: a smaller NA gives a smaller axicon phase gradient, so the same phase discretization error and segment‐boundary compensation error become relatively larger with respect to the phase signal, causing an inward shift of the main‐ring position. As the beam gradually evolves into a stable non‐diffracting form during propagation, the influence of the phase error on the main‐ring position weakens, and the measured radii eventually approach the theoretical values with high accuracy [31, 32].

To quantitatively validate the designed polarization distribution, the transverse Stokes parameters are employed to characterize the polarization state on the main ring. The Stokes parameters are directly calculated from the $E_{x}$ and $E_{y}$ components recorded by FDTD monitors, with their definitions provided in Supporting Information S1: Note S7. The polarization azimuth ψ and ellipticity χ describe, respectively, the variation of the polarization direction with the azimuthal angle and the ellipticity of the polarization ellipse: χ=0 for linear polarization and χ=±π/4 for circular polarization. From the distributions of $s_{1}$, $s_{2}$, $s_{3}$, ψ and χ on the main ring, the designed linear polarization in each sector can be quantitatively verified.

Figure 6a shows the distributions of the normalized Stokes parameters $s_{1}$, $s_{2}$, and $s_{3}$ on the main ring at five propagation planes. It can be seen that, within the designed linear‐polarization sectors, $s_{3}$ is close to 0, indicating that the output is linearly polarized; $s_{1}$ and $s_{2}$ exhibit periodic variations with the azimuthal angle, corresponding to the rotation of the polarization azimuth. Figure 6b presents the distribution of the ellipticity χ and the variation of the polarization azimuth ψ with the azimuthal angle ϕ. Within the target sectors, χ is close to 0, indicating that the polarization state remains linearly polarized. Figure 6c shows the phase distributions of the LCP and RCP components on the main ring, where the LCP and RCP phases exhibit helical distributions. The qualitative polarization ellipse distributions on the main ring are provided in Supporting Information S1: Note S8; combined with the quantitative results of the Stokes parameters, polarization azimuth, and ellipticity, this confirms that the designed metasurface reproduces the target polarization‐order distribution at representative propagation planes.

**FIGURE 6:**
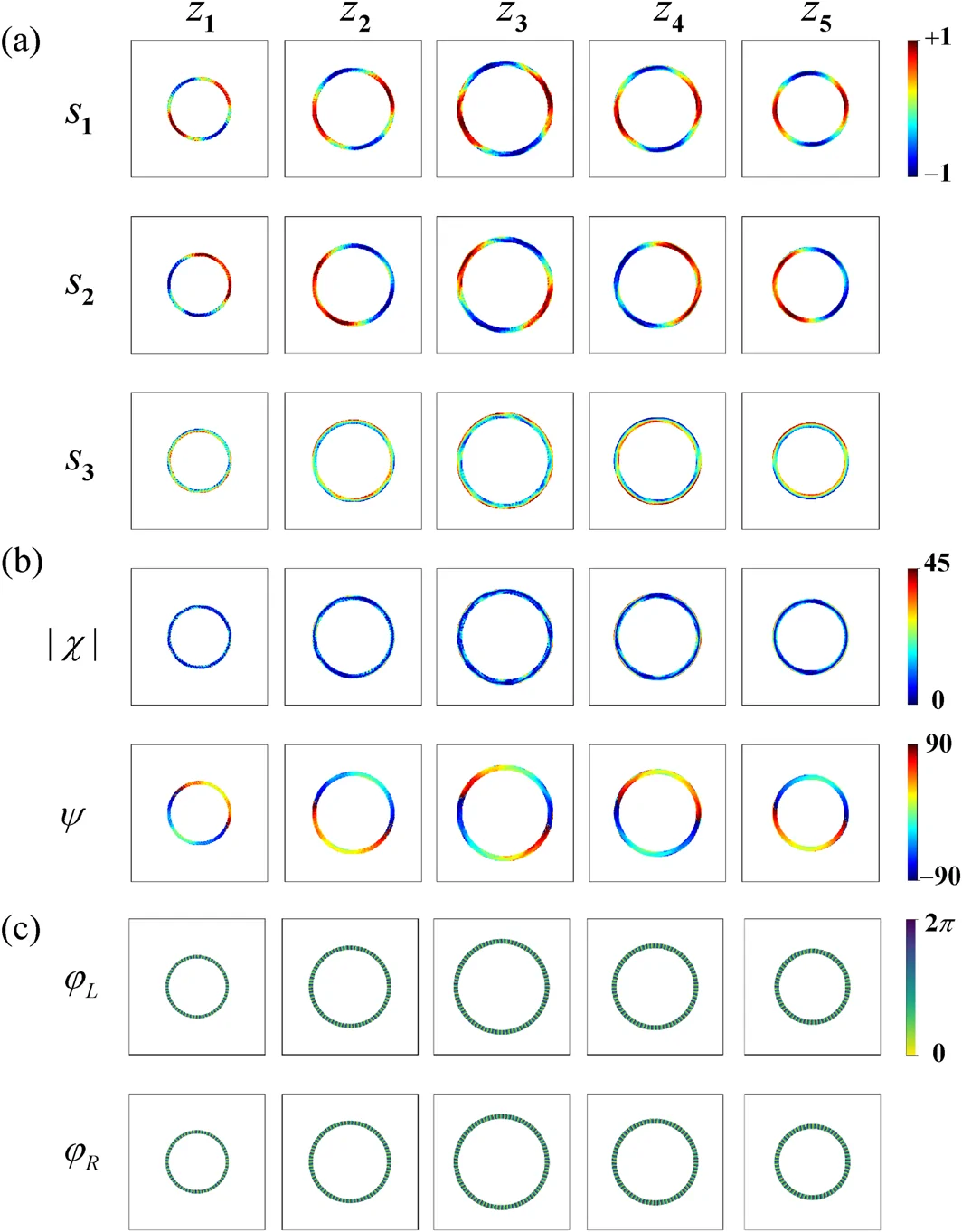
Presents the quantitative polarization characterization of the main ring at five representative propagation planes. From the first to the seventh column, the panels show, in sequence, the normalized Stokes parameters $s_{1}$, $s_{2}$, and $s_{3}$ (ranging from −1 to 1), the absolute value of the ellipticity χ, the polarization azimuth ψ, and the phase distributions of the LCP and RCP components.

The LCP and RCP components are encoded sector‐wise, with different target topological charges assigned to different sectors. Therefore, the target output is not a single global OAM state, but a combination of multiple target OAM orders introduced by the sector‐wise encoding within each spin component. Taking the 69/71 segmented encoding as an example, the two sectors are assigned *l* = 69 and *l* = 71, respectively; owing to the phase reconstruction and sector modulation at the sector boundaries, the target OAM channel consists of three peaks at *l* = 69, *l* = 70, and *l* = 71, where *l* = 70 is the intermediate order between the two sector‐wise target orders. We quantitatively extract the OAM mode purity at different propagation distances and compare it with that of the ideal field. The OAM spectrum analysis is implemented by angular Fourier decomposition within the main‐ring region, where the main‐ring region is defined by the $1/e^{2}$ drop points of the radial intensity profile.

Figure 7 presents a comparison between the simulated and ideal OAM spectra of the LCP and RCP components at five propagation planes. Taking the LCP component at *z* = 42 μm with sector‐wise encoding 69/71 as an example, the actual OAM spectrum exhibits a clear three‐peak structure: *l* = 70 accounts for 0.407, *l* = 69 for 0.282, and *l* = 71 for 0.225. The sum of the three peaks across the four sector‐encoded planes ranges from 82.6% to 91.4%, with an average of 87.79%. At *z* = 69 μm, the single‐OAM‐state design achieves the highest purity, with 89.2% for LCP and 86.8% for RCP. Compared with the ideal field, the main peak positions of the actual spectrum are completely consistent with those of the ideal field, and the three‐peak structure agrees with the segmented encoding design. However, the actual spectrum exhibits certain broadening and low‐intensity sidebands near the target peaks. These sidebands mainly originate from two physical mechanisms: first, phase discretization, that is, the approximation of the continuous phase distribution by a finite number of nanopillar unit cells; second, the boundary effect of segmented encoding, that is, non‐ideal phase compensation at sector boundaries.

**FIGURE 7:**
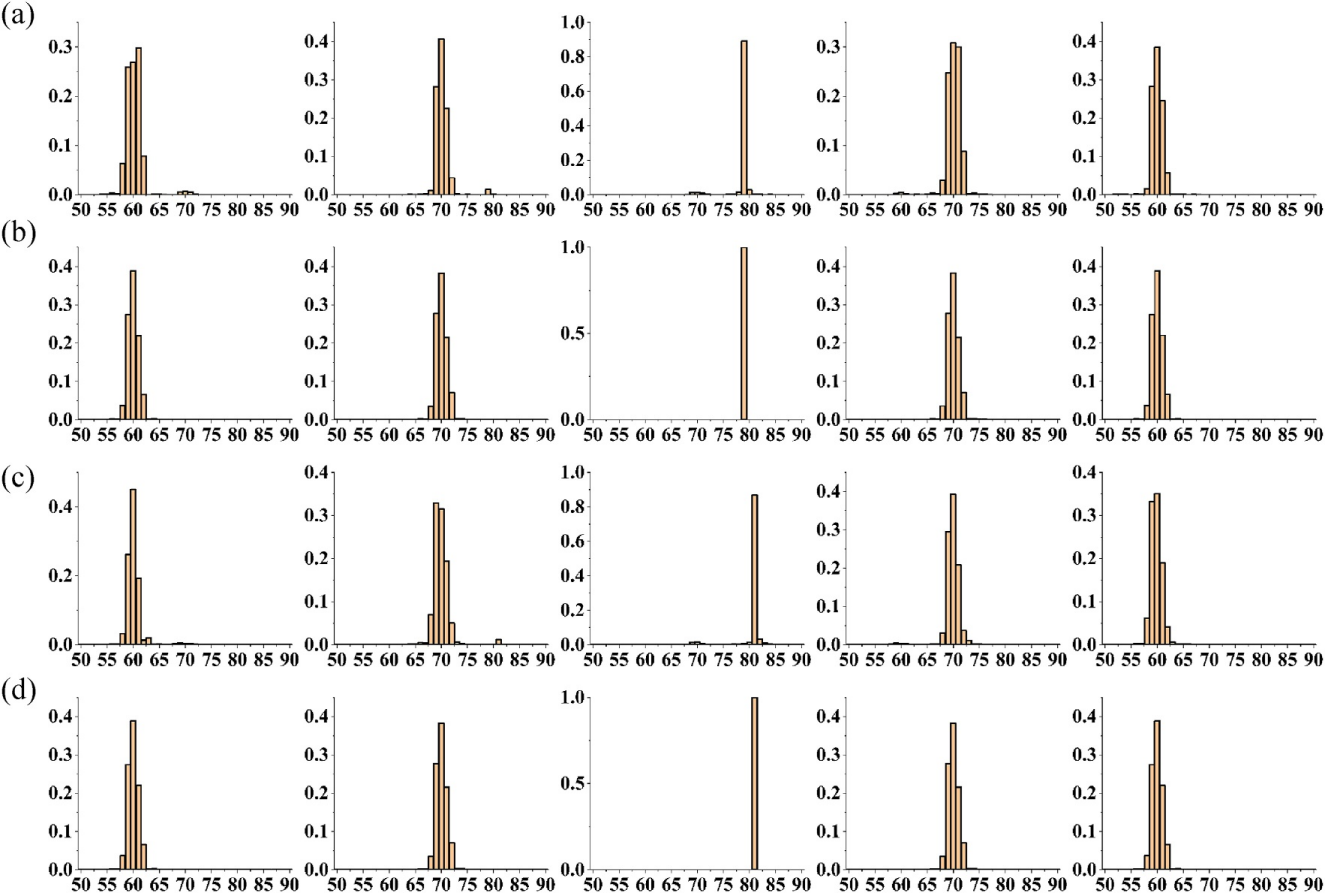
Comparison between the simulated and ideal OAM spectra of the LCP and RCP components at five propagation planes. The horizontal axis denotes the topological charge *l*, and the vertical axis represents the normalized power proportion of each OAM mode. Columns correspond to the five propagation planes $z_{1}$–$z_{5}$, and rows correspond, in order, to the simulated OAM spectrum of the LCP component, the corresponding ideal OAM spectrum, the simulated OAM spectrum of the RCP component, and the corresponding ideal OAM spectrum of the RCP component. The simulated spectra are obtained from full‐wave FDTD simulations, whereas the ideal spectra are calculated from the target phase distribution. (a) The simulated OAM spectrum of the LCP component at the five propagation planes. (b) The corresponding ideal OAM spectrum of the LCP component at the five propagation planes. (c) The simulated OAM spectrum of the RCP component at the five propagation planes. (d) The corresponding ideal OAM spectrum of the RCP component at the five propagation planes.

In the transition region between radial zones, multiple contributions may coexist, and the output field corresponds to a superposition of the target OAM combination states of the two adjacent radial zones. To quantitatively characterize this behavior, the OAM spectra of the region before the transition, the region after the transition, and the transition region itself were extracted separately. The results show that: the OAM spectrum of the region before the transition is dominated by the target peaks designed for that zone; the OAM spectrum of the region after the transition is dominated by another set of target peaks; and in the transition region, both sets of target peaks appear simultaneously, forming a superposition of two OAM combination states. This is consistent with the expected behavior of the piecewise‐continuous design, indicating that the coexistence of multiple components in the transition region is an inherent consequence of the design itself. The OAM spectra of the four transition regions and their two adjacent radial zones were all processed under the same aperture region and the same angular Fourier decomposition with radial integration, so as to ensure comparability. Detailed results are provided in Supporting Information S1: Note S9.

To provide a complete quantitative evaluation, the following metrics are introduced in this work. The total transmittance $T_{\text{MS}}$ is extracted by a power monitor covering the entire metasurface aperture, with a value of 0.702. This value includes the intrinsic absorption of $\text{Ti}O_{2}$, near‐field coupling between nanopillars, finite‐aperture effects, and undesired diffraction orders. The OAM mode purity $\eta_{\text{OAM}}$ is obtained by angular Fourier decomposition of the complex amplitude within the main‐ring region, with an average value of 87.79%. The main‐ring power ratio $\eta_{\text{spatial}}$ is defined as the ratio of the integrated main‐ring intensity to the total intensity within a 40 μm × 40 μm local field of view, ranging from 14.71% to 37.49%. Finally, the global mode conversion efficiency is defined as

(15) $$\eta_{\text{mode}}=T_{\text{MS}}\times \eta_{\text{OAM}}$$

The product gives approximately 61.6% and should be understood as a theoretical composite estimation, representing the scaled fraction of the full‐aperture transmitted energy contained in the target OAM channel within the main‐ring region. This metric does not include the independent contribution of side lobes outside the main ring, nor does it separately distinguish the residual orthogonal‐spin component. The main‐ring power ratio $\eta_{\text{spatial}}$ and the polarization extinction ratio (PER) in the spin–OAM joint domain are provided separately in the main text as complementary metrics. Detailed definitions, sampling methods, and complete data are provided in the Supporting Information S1: Note S10.

To evaluate the polarization separation in the spin–OAM joint domain, we calculate the local polarization extinction ratio (PER) within the same spatial sector and the same topological charge channel:

(16) $$\text{PER}\left(l,\text{sector}\right)=10\text{lo}g_{10}\left(\frac{P_{target\;spin}^{\left(l\right)}}{P_{\text{orthogonal}\;\text{spin}}^{\left(l\right)}}\right)$$

The PER was evaluated at 21 equally spaced radii within the Bessel main‐ring region, and the results are summarized in the Supporting Information S1: Note S11. These results confirm that, in the spin–orbit joint domain, the target spin component dominates in the corresponding OAM channel, while the orthogonal circular polarization component is effectively suppressed and separated. Figure 8 presents the variation of the polarization extinction ratio (PER) with the 21 sampling radii within the main‐ring region at five propagation distances.

**FIGURE 8:**
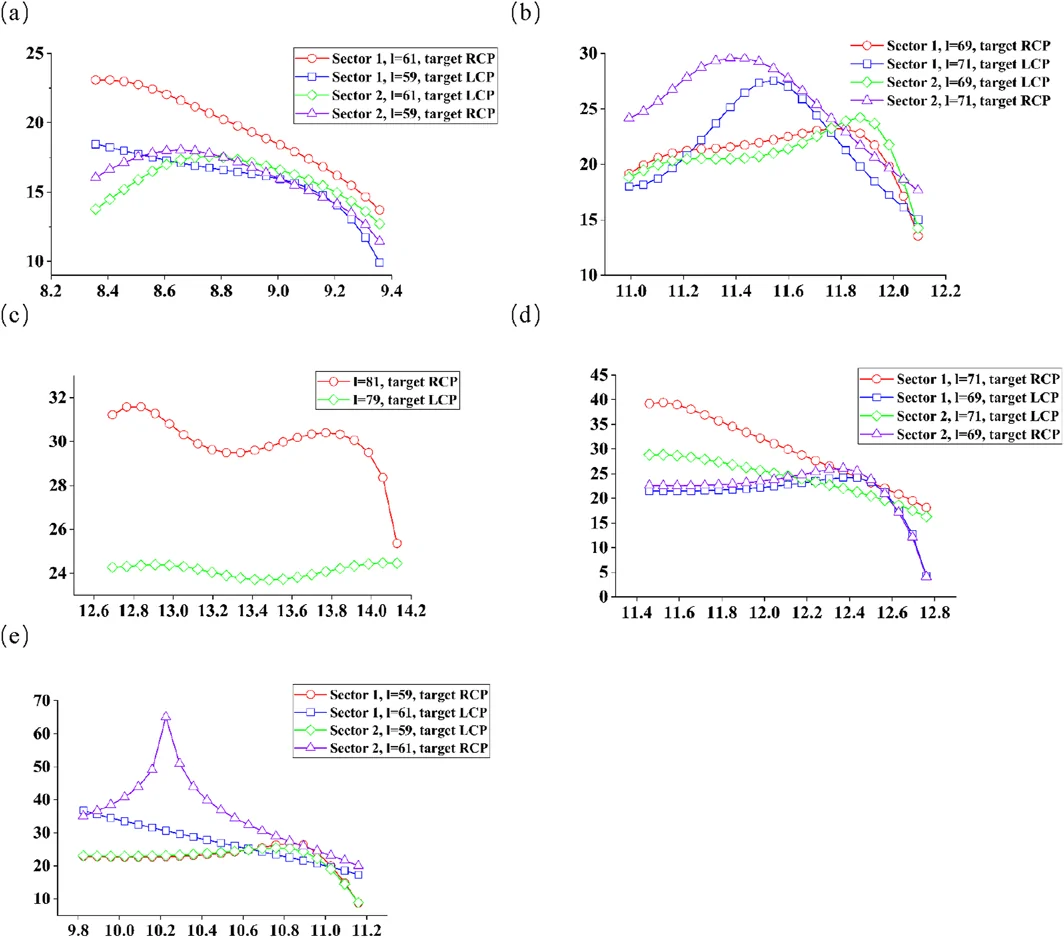
Presents the variation of the polarization extinction ratio (PER) for each channel with the 21 equally spaced sampling radii within the main‐ring region at five propagation distances. The horizontal axis denotes the ring radius in μm, and the vertical axis denotes the PER in dB. (a) through (e) correspond to the variation of the polarization extinction ratio (PER) for each channel at the five different propagation distances, respectively.

To quantitatively describe the actual variations of *L*(*z*) and *P*(*z*), the topological charges *m* and *n* of the LCP and RCP components were extracted by sector‐wise fitting, and *L* = (*m* + *n*)/2 and *P* = (*m*−*n*)/2 were then calculated. At each propagation plane, 720 angular sampling points were taken along the mid‐radius of the main ring to obtain the unwrapped phase *ϕ*(*θ*); for each half‐sector, *ϕ*(*θ*) = *lθ* + *b* was fitted separately, and the fitted slope *l* gives the actual topological charge of that sector.

Figure 9 presents the fitted variations of *L* and *P* with propagation distance *z*. Figure 9a shows the fitted *L* as a function of the propagation distance index (1–15). *L* exhibits a step‐like distribution with plateaus at 60, 70, 80, 70, 60, etc., consistent with the designed sequence. The L plateaus of the two sectors are essentially the same, indicating that the net OAM of the left‐ and right‐circularly polarized components evolves synchronously. Figure 9b shows the difference between the fitted and theoretical *L* for each sector. For both Sector 1 and Sector 2, the differences remain within approximately ± 0.04, confirming that the fitted *L* is highly consistent with the designed *L*. Figure 9c shows the step sequences of the theoretical *P* (upper panel) and the fitted *P* (lower panel). The *p* values of the two sectors switch between +1 and −1, and the fitted values agree well with the theoretical values.

**FIGURE 9:**
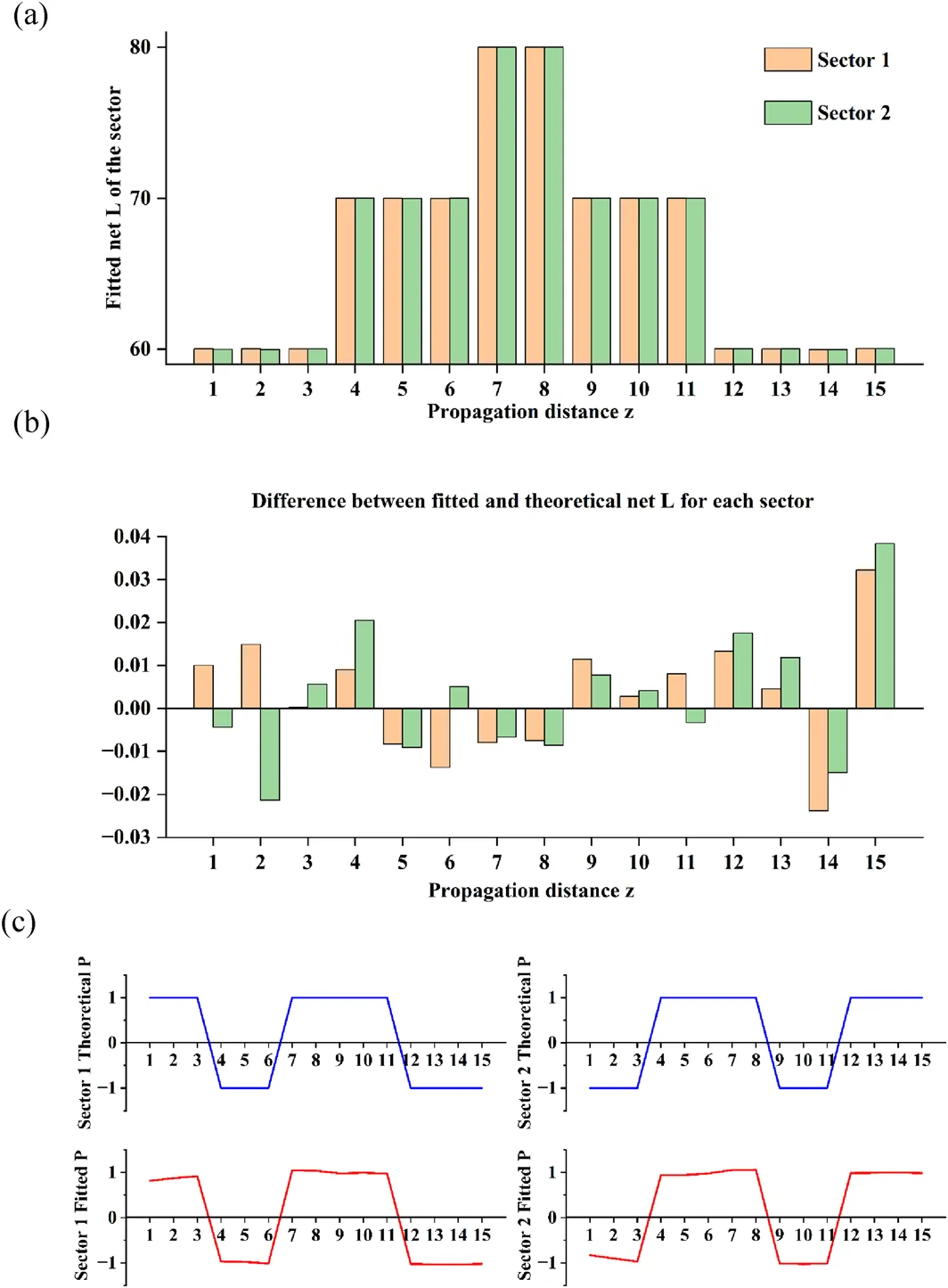
Quantitative verification of the piecewise‐continuous longitudinal evolution of *L*(*z*) and *P*(*z*). (a) Fitted L versus propagation distance index (1–15), showing step‐like plateaus (60, 70, 80, 70, 60, etc.) consistent with the designed sequence. (b) Difference between the fitted and theoretical *L* for each sector, both within approximately ± 0.04. (c) Theoretical (upper) and fitted (lower) *P* step sequences; the *p* values of the two sectors switch between +1 and −1, with the fitted values agreeing with theory.

These results confirm that, within the propagation interval corresponding to each zone, the fitted *L* and *P* agree with the designed values and exhibit a plateau‐like distribution. The fitted L shows a step‐like profile consistent with the designed sequence, with deviations within approximately ± 0.04, while the fitted *P* switches between +1 and −1 in agreement with theory. Therefore, *L*(*z*) and *P*(*z*) in this work are piecewise‐continuous rather than discrete.

## 4 Fabrication Tolerance, Bandwidth, and Aperture Constraints

We further evaluated the robustness of the designed device with respect to fabrication errors, wavelength drift, angular deviation, and large‐aperture fabrication. The results indicate that, under *a* ± 10 nm dimensional deviation of the nanopillars, the main‐ring intensity profile and the target OAM mode remain essentially unchanged, with the target OAM mode purity varying by less than 1%. Under a wavelength drift of ± 10 nm near 633 nm, the OAM purity, main‐ring intensity profile, and three‐peak structure remain stable. In addition to the OAM purity and main‐ring radius, the vector polarization characteristics remain stable under ± 10 nm dimensional deviations and ± 10 nm wavelength drift. The PER profiles under dimensional deviations highly coincide with the baseline; under wavelength drift, $s_{3}$ remains close to 0, and the PER in the central main‐ring region maintains extremely high purity. This indicates good robustness against typical fabrication errors and finite wavelength drift. The device operates normally within an incidence‐angle range of ± 2°. For the highest order designed (*L* = 220), the main ring still falls entirely within the designed aperture of 214 μm × 214 μm. These results indicate that the design is reasonably robust against typical fabrication errors and wavelength drift, while placing certain requirements on the incidence angle and large‐aperture fabrication. Detailed data are provided in Supporting Information S1: Note S12.

## 5 Conclusion

Existing polarization manipulation techniques for vector beams are mostly limited to static transverse polarization control or discrete longitudinal polarization switching. Even when continuous longitudinal manipulation is achievable in some schemes, they are typically restricted to a single polarization order configuration. To overcome this limitation, we propose, in this work, a novel method for generating longitudinally evolving vector beams via a spin‐multiplexed metasurface. This approach enables independent design and synchronized segment‐wise continuous evolution of the net OAM L(z) and polarization order P(z) along the propagation direction, achieving, for the first time, integrated manipulation of nonuniform transverse polarization order distributions and segment‐wise continuous longitudinal evolution of both P and L within a single metasurface.

To realize the above goals, we independently tailor the phase distributions of the two orthogonal circular polarization components and adopt a synergistic encoding strategy combining radial zones and angular sectors, establishing an asymmetric topological charge allocation mechanism. Unlike conventional perfect‐vortex grafting schemes, our method does not require strict coincidence of the main‐ring radii of the two spin components. Instead, by exploiting the inherent property that the radial misalignment between high‐order Bessel beams becomes negligibly small, the two components achieve efficient spatial overlap while maintaining a small radius difference (Δρ≈0.34 μm, relative difference < 4%). This strategy ensures high‐quality vector beam synthesis while preserving the full flexibility of longitudinal optical field manipulation and the excellent non‐diffracting propagation characteristics inherent to Bessel beams.

In summary, the proposed design overcomes the dimensional bottleneck of conventional polarization manipulation techniques for vector beams and extends the technological pathways for multi‐dimensional optical field manipulation, holding significant potential for applications in optical encryption, particle trapping, and high‐capacity optical communications. The main theoretical contributions of this work are as follows. First, it proposes a joint modulation mechanism that combines a transversely nonuniform polarization‐order distribution with the longitudinally piecewise‐continuous evolution of *P*(*z*) and *L*(*z*). Second, it provides a complete phase‐compensation derivation and a spin‐multiplexed Jones matrix design method. Third, it numeric ally demonstrates that high‐order Bessel beams enable efficient same‐scale superposition of different topological charges, which is essential for high‐quality vector beam synthesis. These theoretical results provide clear design guidance and a parameter basis for future experiments.

## Author Contributions

**Lingling Xu:** conceptualization, writing – review and editing, writing – original draft, methodology, visualization, validation, investigation, software. **XueYing Wu:** investigation, visualization. **Mingli Sun:** validation, methodology. **Xiaogang Wang:** project administration, formal analysis. **Xinning Yu:** investigation, validation. **Tingting Sun:** formal analysis, project administration. **Bijun Xu:** funding acquisition, methodology, validation, supervision, resources, data curation.

## Funding

Natural Science Foundation of China (62375245), Ministry of Science and Technology of the People's Republic of China (2022YFC2808203), Youth Fund Project of Zhejiang Provincial Natural Science Foundation (QN26A040009), China Postdoctoral Science Foundation (2025M770832), Zhejiang Provincial Natural Science Foundation of China under Grant (LMS25A040005).

## Conflicts of Interest

The authors declare no conflicts of interest.

## Supporting information


Supporting Information S1

## Data Availability

The data that support the findings of this study are available from the corresponding author upon reasonable request.
