# Supplementary material for: Transverse‐Longitudinal Manipulation of High‐Order Bessel Vector Vortex Beams via Spin‐Multiplexed Metasurfaces
Source: Nanophotonics. 2026 Nov 14;15(22):e70364. doi: 10.1002/nap2.70364 (PMC13768869; doi:10.1002/nap2.70364)
Supplement: Supplementary file 1 [file NAP2-15-e70364-s001.docx]

**Supplementary Material**

**Transverse–Longitudinal Manipulation of High-Order Bessel Vector Vortex Beams via Spin-Multiplexed Metasurfaces**

Lingling Xu, XueYing Wu, Mingli Sun, Xiaogang Wang, Xinning Yu, Tingting Sun, and Bijun Xu*

Department of Physics, Zhejiang University of Science and Technology, Hangzhou 310023, China
E-mail: [xubijun@zust.edu.cn](mailto:xubijun@zust.edu.cn)

**Supplementary Note S1. Asymptotic verification of Bessel zeros and low-/high-order comparison**

**S1.1 Verification of the applicability of the asymptotic formula**

To verify the applicability of the asymptotic formula for the first positive zero of the Bessel function, we systematically compared high-precision numerical solutions with the values calculated from the asymptotic formula. The results are summarized in Table S1.

**Supplementary Table S1.** Comparison between the exact numerical solution and the asymptotic value of the first positive zero of the Bessel function.

| ℓ | Exact solution | Asymptotic value | Absolute difference | Relative Error |
| --- | --- | --- | --- | --- |
| 5 | 8.771484 | 8.173289915 | 0.598194085 | 6.82% |
| 10 | 14.475501 | 13.99809476 | 0.477406239 | 3.30% |
| 20 | 25.417141 | 25.03728375 | 0.379857251 | 1.49% |
| 30 | 36.098337 | 35.76625045 | 0.332086549 | 0.92% |
| 40 | 46.648409 | 46.34657983 | 0.301829171 | 0.65% |
| 50 | 57.116899 | 56.83664587 | 0.280253126 | 0.49% |
| 60 | 67.528786 | 67.26502032 | 0.263765678 | 0.39% |
| 70 | 77.898660 | 77.64808014 | 0.25057986 | 0.32% |
| 80 | 88.235879 | 87.99618952 | 0.239689478 | 0.27% |
| 90 | 98.546847 | 98.31637224 | 0.230474765 | 0.23% |
| 100 | 108.836166 | 108.613634 | 0.222531953 | 0.20% |
| 110 | 119.107265 | 118.8916831 | 0.215581851 | 0.18% |
| 120 | 129.362778 | 129.153352 | 0.209425968 | 0.16% |

This table shows that even for low orders *L*=5, 10, 20, the relative errors between the asymptotic formula and the numerical solution are only 6.82%, 3.30%, and 1.49%, respectively, indicating that the asymptotic formula is also applicable at low orders.

The main ring radius is determined by the first positive zero of the Bessel function:

Therefore, for a fixed topological charge difference (e.g., Δ*L* = 2) at different orders, this paper uses the asymptotic formula to uniformly calculate the corresponding absolute radius difference Δ*ρ* and relative radius difference Δ*ρ*/*ρ*.The relative radius difference is defined as

where is the absolute radius difference, *ρ*(*L*) is the main ring radius corresponding to the central order *L*, and the unit is μm.

**S1.2 Quantitative comparison between low-order and high-order cases**

The absolute radius difference Δ*ρ* corresponds to the main ring misalignment when the topological charge difference between LCP and RCP is 2 (i.e., *P*=±1). The calculation results are summarized in Table S2.

**Supplementary Table S2.** Absolute and relative main-ring radius differences for a fixed topological-charge difference of 2 at different orders.

| ℓ | Δ*ρ* (μm) | Δ*ρ*/*ρ* |
| --- | --- | --- |
| 5 | 0.407396704 | 29.69% |
| 10 | 0.380654486 | 16.20% |
| 20 | 0.364023424 | 8.66% |
| 30 | 0.357336971 | 5.95% |
| 40 | 0.353579734 | 4.54% |
| 50 | 0.351123813 | 3.68% |
| 60 | 0.349371616 | 3.09% |
| 70 | 0.348047659 | 2.67% |
| 80 | 0.347005826 | 2.35% |
| 90 | 0.346160797 | 2.10% |
| 100 | 0.345459128 | 1.89% |
| 110 | 0.344865492 | 1.73% |
| 120 | 0.344355519 | 1.59% |

At low orders *L*=5, 10, and 20, Δ*ρ*/*ρ* is as high as 29.69%, 16.20%, and 8.66%, respectively, indicating that the main rings of the two spin components are clearly separated and the radial mode separation is severe. In contrast, at high orders *L*≥60, the relative radius difference Δ*ρ*/*ρ* drops below 4%, and the main ring radius difference becomes negligible. This quantitatively demonstrates that the proposed method fails at low orders but is effective at high orders.

**S1.3**. **FDTD simulation verification of Δ*ρ* and Δ*ρ*/*ρ* for a practical metasurface**

To verify the above theoretical predictions, we extracted the peak positions of the LCP and RCP main rings from full-wave FDTD simulations of a practical metasurface. The horizontal peak position difference is defined as

$$\Delta x=\left|x_{peak,LCP}-x_{peak,RCP}\right|$$

Here, $x_{peak,LCP}$ and $x_{peak,RCP}$ are the peak center coordinates of the LCP and RCP main rings on the same side in the horizontal intensity profile, respectively. The relative radius difference is defined as Δ*x*/*ρ*.

At five propagation planes, the Δ*x* and Δ*x*/*ρ* extracted from the FDTD simulations are in good agreement with the asymptotic theoretical predictions: at high orders, Δ*ρ*≈0.34 μm, and Δ*ρ*/*ρ*<4%. This directly demonstrates that the theoretical Δ*ρ* is not merely a mathematical approximation but also holds for practical metasurface structures. Δ*ρ* and Δ*x* describe the same physical quantity—the spatial misalignment between the LCP and RCP main rings.

**Supplementary Table S3.** Peak-position differenceΔ*x* and relative radius differenceΔ*x*/*ρ* extracted from full-wave FDTD simulations at five propagation planes.

| z-plane | Δ*x* (+*x* side) | Δ*x* (−*x* side) | Measured main ring radius *ρ* | Δ*x*/*ρ*(+*x* side) | Δ*x*/*ρ*(-*x* side) |
| --- | --- | --- | --- | --- | --- |
| 15 μm | 0.1 | 0.234 | 8.974 | 1.1143% | 2.6075% |
| 42 μm | 0.267 | 0.234 | 11.526 | 2.3165% | 2.0302% |
| 69 μm | 0.267 | 0.267 | 13.561 | 1.9689% | 1.9689% |
| 100 μm | 0.433 | 0.467 | 12.16 | 3.5609% | 3.8405% |
| 125 μm | 0.433 | 0.367 | 10.559 | 4.1008% | 3.4757% |

The asymptotic formula for the first positive zero of the Bessel function is verified to be accurate even at low orders. Using this formula, the absolute radius difference Δ*ρ* and the relative radius difference Δ*ρ*/*ρ* are calculated for a fixed topological-charge difference of 2. At low orders, Δ*ρ*/*ρ* is large, indicating severe radial mode separation; at high orders, Δ*ρ*/*ρ* drops below 4%, and the main-ring radius difference becomes negligible. Full-wave FDTD simulations of a practical metasurface confirm that the theoretical Δ*ρ* and Δ*ρ*/*ρ* are consistent with the simulated values at five propagation planes. These results quantitatively demonstrate that the proposed method is ineffective at low orders but becomes effective at high orders, and that the theoretical Δ*ρ* is not merely a mathematical approximation but also holds for practical metasurface structures.

**Supplementary Note S2: Derivation of the radially and azimuthally piecewise-constant functions**

the phase order $L(\rho)$ is a radially piecewise constant function:

$$L(\rho)=\sum_{i=1}^{N} Li\cdot1_{\left[\rho_{i-1},\rho\left._{i}\right)\right.}(\rho)$$

and the polarization order is a radially–azimuthally jointly piecewise constant function:

Where $P_{i,k}$ is the constant value of the polarization order within the *i*-th annular zone and the *k*-th sector, and the indicator function equals 1 when falls into the *i*-th subregion and 0 otherwise.

**Supplementary Note S3: Phase-offset compensation at sector boundaries**

To eliminate the phase discontinuities at sector boundaries, we introduce a phase‑offset compensation term $\delta_{i,k}$, which depends on both the radial zone index $i$ and the angular sector index *k*. According to Equation (8), the topological charges of the two circular polarization components in the *i*-th radial zone and the *k*-th angular sector are $m_{i,k}=L_{i}+P_{i,k}$ and $n_{i,k}=L_{i}-P_{i,k}$. The corresponding phase‑offset compensations are defined as

$$\begin{matrix} \delta_{i,k}^{(m)}=\frac{2\pi}{M}(\sum_{j=1}^{k-1} m_{i,j}-(k-1)m_{i,k}) \\ \delta_{i,k}^{(n)}=\frac{2\pi}{M}(\sum_{j=1}^{k-1} n_{i,j}-(k-1)n_{i,k}) \end{matrix}$$

Using the symmetric relation between $m_{i,k}$ and $n_{i,k}$ with respect to $L_{i}$, the above compensations can be simplified as

$$\begin{matrix} \delta_{i,k}^{(m)}=\frac{2\pi}{M}(\sum_{j=1}^{k-1} P_{i,j}-(k-1)P_{i,k}) \\ \delta_{i,k}^{(n)}=-\delta_{i,k}^{(m)} \end{matrix}$$

The continuous distribution of this compensation along the radial and angular coordinates is then expressed as

where the compensation term is entirely determined by the distribution of the polarization order $P$ within each sector.

**Supplementary Note S4: Phase design of MS₂**

$MS_{2}$ adopts two independent phase pairs (,) and (,) to generate a Bessel vector vortex beam with two concentric main rings. The inner ring is described by the phases and, employs $NA_{1}=0.6$, and $L$ first increases and then decreases along the radial direction, following the sequence 100, 105, 110, 105, 100. The outer ring is described by the phases and, employs $NA_{2}=0.5$, and $L$ increases monotonically along the radial direction, following the sequence 200, 205, 210, 215, 220. The corresponding phase expressions are:

The target phases and are then obtained by complex superposition and argument extraction:

**Supplementary Note S5.** **Cross-polarization efficiencies of the 12 selected unit cells.**

To evaluate the polarization conversion capability of the selected unit cells, we calculated the cross-polarization efficiency of 12 representative nanopillars. These nanopillars were screened from the metasurface design according to the required phase coverage and transmission performance. The cross-polarization efficiency is defined as the ratio of the power transmitted in the target cross-polarized component to the total incident power. As listed in Supplementary Table S5, the selected unit cells exhibit cross-polarization efficiencies ranging from approximately 0.737 to 0.997, with most values exceeding 0.93. This indicates that the selected nanopillars provide efficient polarization conversion while maintaining the required phase response for the metasurface design.

**Supplementary Table S4. Nanopillars selected by the metasurface design**

| Nanopillar length | Nanopillar width | x-phase | y-phase | x-transmittance | y-transmittance | Cross-polarization efficiency |
| --- | --- | --- | --- | --- | --- | --- |
| 145 | 372 | 0.1156 | 3.5284 | 0.7497 | 0.7527 | 0.7374711 |
| 396 | 68 | 1.0393 | 3.8825 | 0.9719 | 0.9077 | 0.9190352 |
| 322 | 92 | 1.3671 | 4.4192 | 0.9811 | 0.9632 | 0.9702048 |
| 301 | 114 | 1.8885 | 5.0742 | 0.9958 | 0.9993 | 0.9970649 |
| 319 | 124 | 2.4118 | 5.7933 | 0.9988 | 0.9904 | 0.9803573 |
| 294 | 138 | 2.9629 | 5.8272 | 0.9879 | 0.9921 | 0.9710911 |
| 372 | 145 | 3.5284 | 6.3988 | 0.7527 | 0.7497 | 0.7374726 |
| 75 | 375 | 3.9923 | 7.3998 | 0.9231 | 0.9735 | 0.9316357 |
| 82 | 386 | 4.5175 | 7.5515 | 0.967 | 0.9774 | 0.9693891 |
| 110 | 308 | 5.0377 | 8.0795 | 0.9985 | 0.9938 | 0.993672 |
| 117 | 326 | 5.5784 | 8.4283 | 0.9993 | 0.9996 | 0.9783409 |
| 138 | 301 | 6.0991 | 9.2933 | 0.9493 | 0.9866 | 0.9672804 |

**Supplementary Note S6: Definitions of Spatial Overlap Metrics**

To quantitatively evaluate the spatial overlap between the LCP and RCP components within the main-ring region, three metrics are defined.

The peak position difference $\Delta x$ is defined as follows. The peak center coordinates on the same side (left or right) are extracted from the horizontal profiles of the LCP and RCP components, respectively, and the absolute difference

$$\Delta x=\left|x_{peak,L}-x_{peak,R}\right|$$

is calculated to directly quantify the lateral shift between the two main rings. The common overlap ratio OR is defined as follows. Let $w_{L}$ and $w_{R}$ be the $1/e^{2}$ width of the LCP and RCP main rings, respectively, and let $w_{com}$ be the width of their common interval. The overlap ratios for the two components are then given by

$$OR_{L}=w_{com}/w_{L}$$

$$OR_{R}=w_{com}/w_{R}$$

The smaller of the two values is taken as the common overlap ratio $OR$ for that half ring. The common overlap ratio reflects the proportion of the main ring that is jointly covered by both the LCP and RCP components.

The matching factor MF is evaluated over the overlapping radial interval $\left[x_{left}\right.,\left.x_{right}\right]$, defined as the intersection of the $1/e^{2}$ widths of the LCP and RCP main rings. The normalized overlap integral is then given by

where $I_{L}(x)$ and $I_{R}(x)$ are the intensity distributions of the LCP and RCP components along the horizontal profile, respectively. This metric ranges from 0 to 1.A value of MF close to 1 indicates that the two components exhibit nearly identical intensity profiles within the main-ring region, corresponding to excellent spatial overlap. Conversely, a smaller MF signifies poorer spatial overlap between the two components.

Horizontal intensity profiles are extracted along the *x*-axis at *y*=0. For each spin component, the left and right main-ring peaks are identified from the corresponding horizontal profile. All spatial overlap metrics are calculated separately for the *x*<0 and *x*>0 sides, so that the left and right half-rings are evaluated independently.

It should be emphasized that Δ*x*, OR, and MF are spatial overlap metrics. They quantify how well the intensity distributions of the LCP and RCP components coincide in the transverse plane. They do not represent the polarization purity of the synthesized vector beam.These metrics are calculated separately for the *x*<0 and *x*>0 sides at each observation plane.

**Supplementary Note S7. Definitions of transverse Stokes parameters, polarization azimuth, and ellipticity**

To quantitatively validate the designed polarization distribution, we introduce the transverse Stokes parameters. For a transverse electric field, the Stokes parameters are defined as:

The normalized Stokes parameters are given by. The polarization azimuth and ellipticity are respectively expressed as

Here, describes the variation of the polarization direction with the azimuthal angle, and describes the ellipticity of the polarization ellipse. For linear polarization,; for circular polarization,. The Stokes parameters are directly calculated from the Ex and Ey components recorded by the FDTD field monitors.

**Supplementary Note S8. Polarization ellipses**

Supplementary Figure S1 presents the polarization ellipse distributions on the main ring at five representative propagation planes *z*_1_–*z*_5_. The five panels correspond to the five propagation planes, and each panel shows the polarization ellipses at several sampling points on the main ring. It can be observed that: the major-axis orientation of the polarization ellipses varies continuously with the azimuthal angle, indicating that the polarization azimuth *ψ*(*ϕ*) varies continuously with *ϕ*; the orientation variation of the ellipse major axis differs among different sectors, showing opposite trends in the sectors with $P=+1$ and $P=-1$, consistent with the designed polarization orders;within the designed linear-polarization sectors, the ellipses degenerate into nearly straight lines, indicating that the polarization state remains linearly polarized, consistent with;the ellipse orientations differ among different propagation planes, indicating that the polarization state evolves with propagation distance, consistent with the segmented longitudinal design.

Therefore, the polarization ellipse figure qualitatively demonstrates the spatially varying polarization-order distribution and the longitudinal polarization evolution on the main ring. Combined with the quantitative results of the Stokes parameters, polarization azimuth, and ellipticity, this confirms that the designed metasurface faithfully reproduces the target spatially varying polarization-order distribution at representative propagation planes.

Figure S1 Polarization ellipse distributions on the main ring at five representative propagation planes *z*_1_–*z*_5_. The five panels correspond to the five propagation planes, and each panel shows the polarization ellipses at several sampling points on the main ring.

**Supplementary Note S9. OAM spectral behavior in the transition regions between radial zones**

In the piecewise-continuous design, the radial domain is divided into several annular zones, each independently encoding a target OAM combination state. Transition regions exist between adjacent annular zones. In these transition regions, multiple contributions may coexist, and the output field no longer corresponds to a single pure OAM state, but rather a superposition of the target OAM combination states of the two adjacent radial zones. To quantitatively characterize this behavior, this note extracts the OAM spectra of the region before the transition, the region after the transition, and the transition region itself, and compares them.

To avoid artifacts caused by different apertures, the four transition regions and their adjacent radial zones were all processed with the same aperture region, the same number of angular sampling points, and the same radial integration range.

**Supplementary Table S5. Propagation planes of the four transition regions and their adjacent radial zones.**

| Transition region | Z_before | Z_transition | Z_after | Radial integration aperture |
| --- | --- | --- | --- | --- |
| 1 | 15 μm | 22 μm | 42 μm | 0–12 μm |
| 2 | 42 μm | 55 μm | 69 μm | 0–14 μm |
| 3 | 69 μm | 86 μm | 100 μm | 0–15 μm |
| 4 | 100 μm | 114 μm | 125 μm | 0–15 μm |

Supplementary **Figure S2 presents the OAM spectra of the four transition regions and their adjacent radial zones. The rows correspond to the four transition regions at *z* = 22, 55, 86, and 114 μm, while the columns correspond to the region before the transition, the transition region itself, and the region after the transition. In each panel, the horizontal axis denotes the topological charge *l*, and the vertical axis denotes the normalized power proportion. These results are consistent with the expected behavior of the piecewise-continuous design. Because each radial zone independently encodes a target OAM combination state, the target peaks of two adjacent zones necessarily coexist in the transition region, forming a superposition of two OAM combination states. The coexistence of multiple components in the transition region is therefore an inherent consequence of the design itself, rather than numerical noise or a design flaw.**

Figure S2 OAM spectra of the four transition regions and their adjacent radial zones. Rows correspond to the four transition regions at *z*=22,55,86 and 114 μm; columns correspond to the region before the transition, the transition region itself, and the region after the transition. The horizontal axis denotes the topological charge *l*, and the vertical axis denotes the normalized power proportion.

**Supplementary Note S10: Quantitative Performance Metrics**

To comprehensively and quantitatively evaluate the performance of the proposed metasurface, five metrics are introduced in this work: the cross-polarization efficiency *η*_cross_, the total transmittance T_MS_, the OAM mode purity *η*_OAM_, the main-ring power ratio *η*_spatial_, and the global mode conversion efficiency *η*_mode_. The definitions, analysis ranges, and results of each metric are summarized in the Supplementary **Table S6.**

**Supplementary Tables S6 The definitions, analysis ranges, and results of Quantitative Performance Metrics**

| Metric | Physical meaning | Analysis method / monitor | Result |
| --- | --- | --- | --- |
| $\eta_{cross}$ | Unit-cell cross-polarization efficiency, i.e., the intrinsic efficiency of converting incident LCP into RCP | Jones matrix analysis; 12 selected nanopillars | 73.7%–99.7%, average ≈93.9% |
| $T_{MS}$ | Total transmittance of the metasurface, characterizing the global light-throughput capability | FDTD power monitor covering the full aperture of 214 μm × 214 μm | 0.702 |
| $\eta_{OAM}$ | Target OAM channel purity | Global OAM spectrum analysis in the main-ring region and sector-wise phase-gradient analysis | Average 87.79%, range 82.6%–91.4% |
| $\eta_{spatial}$ | Local power fraction of the main ring, characterizing the beam “shape quality” | Integrated intensity fraction of the main ring within a 40 μm × 40 μm local field of view | For the five planes: 14.71%, 27.19%, 37.49%, 32.47%, and 30.68%, respectively |
| $\eta_{mode}$ | a theoretical composite estimation, representing the scaled fraction of the full-aperture transmitted energy contained in the target OAM channel within the main-ring region. | $\eta_{mode}=T_{MS}\times\eta_{OAM}$ | Approximately  0.702×87.79%≈61.6% |

Specifically, the cross-polarization efficiency ($\eta_{cross}$) is calculated through Jones matrix analysis. This design is based on the geometric phase principle of a near-half-wave plate: the phase difference between the *x* and *y* directions of the nanopillar,, is close to π, which causes the spin state of the incident circularly polarized light to flip during transmission, while simultaneously acquiring a geometric phase related to the in-plane orientation angle of the nanopillar. According to Jones matrix analysis, the cross-polarization (LCP→RCP) conversion efficiency of each nanopillar is:

Here, $T_{avg}=(T_{x}+T_{y})/2$ is the average transmittance, and is the phase difference between the *x* and *y* directions. Calculations for 12 selected nanopillars show that their cross-polarization efficiencies range from 73.7% to 99.7%, with an average cross-polarization efficiency of approximately 93.9%.

The total transmittance $T_{MS}$ was directly extracted using an FDTD power monitor covering the entire metasurface aperture (214 μm×214 μm), yielding a value of 0.702. This value fully incorporates the real energy losses caused by the intrinsic absorption of the TiO₂ material, near-field coupling effects between nanopillar units, the finite aperture of the metasurface, and undesired diffraction orders. In the unit-cell simulations, the average transmittance of the selected nanopillars was approximately 0.93; after arranging them into a 511×511 metasurface array, the transmittance decreased to 0.702. This approximately 20% relative loss quantifies the combined influence of the aforementioned non-ideal factors when transitioning from an “ideal unit cell” to a “practical array.”

The OAM mode purity $\eta_{OAM}$ was characterized by two complementary methods: first, the full angular Fourier transform of the complex amplitude within the main ring region, i.e., global OAM spectrum analysis; and second, sector-wise phase gradient fitting. Taking the LCP component at *z*=42 μm with segmented encoding 69/71 as an example, the global OAM spectrum exhibits a clear three-peak structure:*l*=70, proportion = 0.407,*l*=69, proportion = 0.282,*l*=71, proportion = 0.225.

The sum of the three peaks across the four segmented encoding planes ranges from 82.6% to 91.4%. At z=69 μm, the single-OAM-state design achieves the highest purity, with 89.2% for LCP and 86.8% for RCP. The average $\eta_{OAM}$ over the 10 data sets is 87.79%. A detailed description of the OAM spectrum analysis method is provided in the Supplementary Note S13. Supplementary Tables S7 and S8 respectively report the proportions of global OAM spectra showing a pronounced three-peak structure and summarize the sector-wise phase-gradient fitting results.

The main-ring power ratio $\eta_{spatial}$ is defined as the ratio of the integrated intensity of the main ring to the total integrated intensity within a 40 μm×40 μm local field of view. This metric characterizes the local intensity contrast of the main ring relative to the approximately 12–18 adjacent side lobes and is a characteristic quantity of the beam’s “shape quality,” rather than a measure of global energy concentration. The reason for choosing a 40 μm field of view instead of the global aperture is that the side lobes of higher-order Bessel beams extend very far radially; if the global aperture were used, $\eta_{spatial}$ would depend strongly on the monitor size and thus could not serve as an intrinsic characteristic parameter of the beam. A 40 μm field of view exactly covers the main ring and its adjacent side lobes, providing a reproducible local shape-quality metric. Among the data at the five planes, $\eta_{spatial}$=14.71% at z=15 μm, which is the lowest, indicating that in the early propagation stage the energy is more dispersed and there are more side lobes; the remaining values fall within 30%–40%, indicating that the main ring maintains good and stable concentration at most propagation distances.

Finally, the global mode conversion efficiency is defined as

$$\eta_{mode}=T_{MS}\times\eta_{OAM}$$

The product gives approximately 61.6% and should be understood as a theoretical composite estimation, representing the scaled fraction of the full-aperture transmitted energy contained in the target OAM channel within the main-ring region. This metric does not include the independent contribution of side lobes outside the main ring, nor does it separately distinguish the residual orthogonal-spin component. The main-ring power ratio $\eta_{spatial}$ and the polarization extinction ratio (PER) in the spin–OAM joint domain are provided separately in the main text as complementary metrics. Together, these three metrics quantify what fraction of the incident power is converted into the desired vector beam.

**Supplementary Table S7.** OAM mode purity at five propagation planes.

| Propagation distance | LCP three-peak structure | Sum of the three peaks | RCP three-peak structure | Sum of the three peaks | *L* |
| --- | --- | --- | --- | --- | --- |
| 15 μm | 59(0.259)  60(0.269)  61(0.298) | 82.60% | 59(0.261)  60(0.450)  61(0.193) | 90.40% | 60 |
| 42 μm | 69(0.282)  70(0.407)  71(0.225) | 91.40% | 69(0.329)  70(0.315)  71(0.194) | 83.80% | 70 |
| 69 μm | 79(0.892) | 89.20% | 81(0.868) | 86.80% | 80 |
| 100 μm | 69(0.247)  70(0.308)  71(0.300) | 85.50% | 69(0.295)  70(0.393)  71(0.208) | 89.60% | 70 |
| 125 μm | 59(0.283)  60(0.385)  61(0.245) | 91.30% | 59(0.332)  60(0.351)  61(0.190) | 87.30% | 60 |

**Supplementary Table S8.** Summary of sector-wise phase-gradient fitting results for five propagation planes and two polarization components (LCP and RCP).

| Propagation distance | Component | Sector 1 design value | fitted value Sector  1 | Mean instantaneous slope | Sector 2 design value | fitted value Sector  2 | Mean instantaneous slope |
| --- | --- | --- | --- | --- | --- | --- | --- |
| 15 | lcp | 59 | 59.22289 | 59.58 | 61 | 60.79490 | 60.42 |
| 15 | rcp | 61 | 60.81871 | 60.55 | 59 | 59.16384 | 59.42 |
| 42 | lcp | 71 | 70.91778 | 70.77 | 69 | 69.08226 | 69.23 |
| 42 | rcp | 69 | 69.05236 | 69.26 | 71 | 70.88058 | 70.74 |
| 69 | lcp | 79 | 78.95501 | 79.04 | 79 | 78.95460 | 78.97 |
| 69 | rcp | 81 | 81.03984 | 81.01 | 81 | 81.03584 | 81.00 |
| 100 | lcp | 69 | 69.05987 | 69.23 | 71 | 70.96912 | 70.77 |
| 100 | rcp | 71 | 70.94414 | 70.80 | 69 | 69.03433 | 69.20 |
| 125 | lcp | 61 | 60.98824 | 60.83 | 59 | 59.05912 | 59.17 |
| 125 | rcp | 59 | 59.02734 | 59.18 | 61 | 60.96567 | 60.82 |

**Supplementary Note S11.** **polarization extinction ratio (PER)**

To avoid numerical contingency introduced by single-radius sampling, we adopted the following sampling strategy: first, the inner and outer boundaries of the Bessel ring, Rinner and Router, were automatically detected and defined using a threshold of 20% of the peak intensity in the horizontal profile of the total intensity (RCP + LCP); then, 21 equally spaced radii were sampled within the ring, and spiral harmonic decomposition was performed independently at each radius to calculate the PER; finally, the reported value is the average over the central region of the ring, excluding the outer 20% margin, so as to avoid numerical noise in low-signal-to-noise regions. This “multi-point sampling + central-region averaging” strategy not only ensures the physical reliability of the PER extraction, since the OAM information of a Bessel beam is concentrated in the main lobe of the ring, but also effectively avoids the sensitivity of a single radius to alignment errors.

**Supplementary Table S9** **the average polarization extinction ratio (PER) for each channel at different propagation distances.**

| z=15 μm | 19.46086143 | 15.89729905 | 15.89883857 | 16.07476429 |
| --- | --- | --- | --- | --- |
| z=42 μm | 21.05393476 | 21.70333381 | 20.87516429 | 25.21062286 |
| z=69 μm | 29.96494952 | 24.13281619 | / | / |
| z=95 μm | 29.80970857 | 20.87359952 | 24.00665333 | 21.85131905 |
| z=125 μm | 22.56772143 | 27.00023095 | 22.35106619 | 35.69976381 |

The above results indicate that, in the spin–orbit joint domain, the target spin dominates in the corresponding OAM channel, while the orthogonal circular polarization component is effectively suppressed and separated. **Detailed data are provided in Supplementary Table S10.**

**Supplementary Table S10.** **The polarization extinction ratio (PER) for each channel at 5 propagation distances.**

| 15 μm | S_1_, *l*=61,target RCP | S_1_, *l*=59,target LCP | S_2_, *l*=61,target LCP | S_2_, *l*=59,target RCP |
| --- | --- | --- | --- | --- |
| 8.35696 | 23.0881 | 18.4581 | 13.77171 | 16.06053 |
| 8.40701 | 23.08916 | 18.22716 | 14.48084 | 16.6368 |
| 8.45705 | 22.97942 | 17.9846 | 15.19966 | 17.13138 |
| 8.50709 | 22.75698 | 17.74258 | 15.88789 | 17.5283 |
| 8.55713 | 22.43781 | 17.50964 | 16.50021 | 17.81513 |
| 8.60717 | 22.04752 | 17.2915 | 16.99363 | 17.98381 |
| 8.65721 | 21.61306 | 17.09152 | 17.3384 | 18.0313 |
| 8.70726 | 21.15683 | 16.91073 | 17.52632 | 17.96057 |
| 8.7573 | 20.69437 | 16.7478 | 17.57111 | 17.78135 |
| 8.80734 | 20.23423 | 16.59866 | 17.50054 | 17.51002 |
| 8.85738 | 19.77906 | 16.45611 | 17.34543 | 17.16787 |
| 8.90742 | 19.32685 | 16.30916 | 17.1313 | 16.77798 |
| 8.95746 | 18.87197 | 16.14229 | 16.87458 | 16.36157 |
| 9.00751 | 18.40605 | 15.93466 | 16.58222 | 15.93469 |
| 9.05755 | 17.91855 | 15.65917 | 16.25305 | 15.50554 |
| 9.10759 | 17.39705 | 15.28157 | 15.87931 | 15.07207 |
| 9.15763 | 16.82752 | 14.75942 | 15.44799 | 14.61888 |
| 9.20767 | 16.19425 | 14.04029 | 14.94137 | 14.11235 |
| 9.25771 | 15.47952 | 13.05814 | 14.33677 | 13.49201 |
| 9.30776 | 14.66256 | 11.7251 | 13.60518 | 12.65549 |
| 9.3578 | 13.71723 | 9.91508 | 12.7081 | 11.43241 |

| 42 μm | S_1_, *l*=69,target RCP | S_1_, *l*=71,target LCP | S_2_, *l*=69,target LCP | S_2_, *l*=71,target RCP |
| --- | --- | --- | --- | --- |
| 10.99249 | 19.15442 | 18.00925 | 18.82878 | 24.16533 |
| 11.04754 | 19.9569 | 18.16524 | 19.46998 | 24.76345 |
| 11.10259 | 20.59258 | 18.73405 | 19.99118 | 25.6698 |
| 11.15763 | 21.0148 | 19.63842 | 20.33058 | 26.75484 |
| 11.21268 | 21.25073 | 20.81385 | 20.48741 | 27.84237 |
| 11.26772 | 21.37499 | 22.19203 | 20.51628 | 28.74325 |
| 11.32277 | 21.46598 | 23.68435 | 20.4946 | 29.33007 |
| 11.37781 | 21.57898 | 25.16515 | 20.49227 | 29.58467 |
| 11.43286 | 21.74099 | 26.45736 | 20.55892 | 29.5483 |
| 11.48791 | 21.95648 | 27.33207 | 20.7245 | 29.23995 |
| 11.54295 | 22.21537 | 27.55804 | 21.00453 | 28.64302 |
| 11.598 | 22.49979 | 27.03483 | 21.40487 | 27.75791 |
| 11.65304 | 22.78731 | 25.89525 | 21.92385 | 26.64426 |
| 11.70809 | 23.04712 | 24.40968 | 22.54925 | 25.40354 |
| 11.76314 | 23.22393 | 22.81678 | 23.24291 | 24.13309 |
| 11.81818 | 23.20643 | 21.25922 | 23.89448 | 22.89907 |
| 11.87323 | 22.79624 | 19.80168 | 24.21835 | 21.73405 |
| 11.92827 | 21.73789 | 18.46263 | 23.6725 | 20.64512 |
| 11.98332 | 19.85559 | 17.23496 | 21.76597 | 19.62157 |
| 12.03837 | 17.13441 | 16.0959 | 18.55644 | 18.6391 |
| 12.09341 | 13.5417 | 15.00927 | 14.2508 | 17.66032 |

| 100 μm | S_1_, *l*=71,target RCP | S_1_, *l*=69,target LCP | S_2_, *l*=71,target LCP | S_2_, *l*=69,target RCP |
| --- | --- | --- | --- | --- |
| 11.45955 | 39.20466 | 21.48194 | 28.8229 | 22.59504 |
| 11.5246 | 39.45147 | 21.47111 | 28.83408 | 22.53827 |
| 11.58966 | 38.98885 | 21.47877 | 28.67975 | 22.51987 |
| 11.65471 | 38.05716 | 21.50915 | 28.36049 | 22.54395 |
| 11.71977 | 36.92615 | 21.56648 | 27.91324 | 22.61462 |
| 11.78482 | 35.74349 | 21.65509 | 27.38637 | 22.7361 |
| 11.84987 | 34.56173 | 21.7797 | 26.82108 | 22.91309 |
| 11.91493 | 33.39144 | 21.94509 | 26.24416 | 23.15044 |
| 11.97998 | 32.23077 | 22.1565 | 25.66835 | 23.45373 |
| 12.04504 | 31.0772 | 22.41886 | 25.09573 | 23.82821 |
| 12.11009 | 29.93036 | 22.73601 | 24.52142 | 24.27708 |
| 12.17515 | 28.79134 | 23.10764 | 23.93655 | 24.79513 |
| 12.2402 | 27.66104 | 23.52198 | 23.33035 | 25.35178 |
| 12.30525 | 26.5391 | 23.93771 | 22.69177 | 25.85009 |
| 12.37031 | 25.4228 | 24.24245 | 22.01024 | 26.04769 |
| 12.43536 | 24.30627 | 24.17998 | 21.27568 | 25.50686 |
| 12.50042 | 23.17983 | 23.31331 | 20.47773 | 23.8175 |
| 12.56547 | 22.02865 | 21.21872 | 19.60394 | 20.97161 |
| 12.63053 | 20.83027 | 17.74923 | 18.63642 | 17.14955 |
| 12.69558 | 19.54997 | 12.66955 | 17.54628 | 12.11135 |
| 12.76063 | 18.13133 | 4.20632 | 16.28319 | 4.10574 |

| 125 μm | S_1_, *l*=59,target RCP | S_1_, *l*=61,target LCP | S_2_, *l*=59,target LCP | S_2_, *l*=61,target RCP |
| --- | --- | --- | --- | --- |
| 9.82485 | 22.8992 | 36.79356 | 23.08378 | 35.04144 |
| 9.89158 | 22.78321 | 35.57172 | 22.98103 | 36.63097 |
| 9.9583 | 22.69653 | 34.4915 | 22.91663 | 38.47291 |
| 10.02502 | 22.64453 | 33.4844 | 22.89362 | 40.75934 |
| 10.09174 | 22.63245 | 32.51168 | 22.91452 | 43.9014 |
| 10.15847 | 22.66565 | 31.55397 | 22.98138 | 49.09853 |
| 10.22519 | 22.74984 | 30.60359 | 23.09607 | 65.07874 |
| 10.29191 | 22.89149 | 29.66024 | 23.2603 | 50.98989 |
| 10.35863 | 23.09845 | 28.72669 | 23.47561 | 43.9695 |
| 10.42535 | 23.38057 | 27.80591 | 23.74305 | 39.86442 |
| 10.49208 | 23.75031 | 26.89954 | 24.06199 | 36.87215 |
| 10.5588 | 24.22277 | 26.00715 | 24.42693 | 34.4669 |
| 10.62552 | 24.81302 | 25.12602 | 24.81972 | 32.42065 |
| 10.69224 | 25.52289 | 24.25116 | 25.19109 | 30.61157 |
| 10.75897 | 26.29062 | 23.37535 | 25.42168 | 28.96446 |
| 10.82569 | 26.83055 | 22.48906 | 25.26171 | 27.42682 |
| 10.89241 | 26.36312 | 21.58003 | 24.31948 | 25.95741 |
| 10.95913 | 24.02272 | 20.6323 | 22.24915 | 24.51958 |
| 11.02585 | 20.02255 | 19.62439 | 18.97123 | 23.07621 |
| 11.09258 | 14.8999 | 18.52584 | 14.47905 | 21.58438 |
| 11.1593 | 8.74178 | 17.29075 | 8.82437 | 19.98777 |

| 69 μm | *l*=81, target RCP | *l*=79, target LCP |
| --- | --- | --- |
| 12.69391 | 31.2242 | 24.27203 |
| 12.76564 | 31.58168 | 24.30938 |
| 12.83736 | 31.58884 | 24.35711 |
| 12.90909 | 31.28286 | 24.38539 |
| 12.98082 | 30.80748 | 24.37069 |
| 13.05254 | 30.31526 | 24.30224 |
| 13.12427 | 29.90787 | 24.18611 |
| 13.196 | 29.63284 | 24.04308 |
| 13.26772 | 29.50039 | 23.90085 |
| 13.33945 | 29.49846 | 23.7854 |
| 13.41118 | 29.60178 | 23.71519 |
| 13.4829 | 29.77688 | 23.69911 |
| 13.55463 | 29.98533 | 23.73698 |
| 13.62636 | 30.18641 | 23.82129 |
| 13.69808 | 30.33909 | 23.93926 |
| 13.76981 | 30.40242 | 24.07502 |
| 13.84153 | 30.33216 | 24.21148 |
| 13.91326 | 30.06972 | 24.33199 |
| 13.98499 | 29.50835 | 24.4215 |
| 14.05671 | 28.36046 | 24.46711 |
| 14.12844 | 25.36146 | 24.45793 |

**Supplementary Note S12.Fabrication Tolerance, Bandwidth, and Aperture Constraints**

**Fabrication tolerance.**The effect of simultaneously increasing both the length and width of the TiO₂ nanopillars by 10 nm was evaluated. The results show that the main-ring intensity profile remains essentially unchanged. Supplementary Table S11 compares the main-ring radius errors between the +10 nm sample and the original design at five propagation planes. The errors for the original design are 15.41%, 6.41%, 3.28%, 1.27%, and 0.47%, respectively; the corresponding errors for the +10 nm sample are 15.26%, 6.28%, 3.27%, 1.13%, and 0.47%, respectively. The two sets of results almost completely overlap, with a maximum difference of only about 0.15%. The OAM purity remains essentially unchanged, and the OAM spectrum of the sector-encoded plane still retains a clear three-peak structure, with a maximum variation in the total proportion of the three peaks of about 0.8% (Supplementary Table S12). The total transmittance changes from 0.702 to 0.681, with a variation of less than 3%.

We further increased both the length and width of each nanopillar unit in the original design by 10 nm and arranged them in the original order, and extracted the x-phase and y-phase sequences of the +10 nm sample. In Supplementary Figure S3 , the x-phase and y-phase of the original design are represented by purple squares (phix) and blue upward triangles (phiy), respectively; the x-phase and y-phase of the +10 nm sample are represented by red circles (xphase) and green downward triangles (yphase), respectively. The horizontal axis is the nanopillar index, and the vertical axis is the phase (in rad). The results show that the variation trends of the x-phase and y-phase with the nanopillar index in the +10 nm sample are consistent with those of the original design, with no phase jumps or anomalies. However, the maximum phase difference between the +10 nm sample and the original design is about 0.995 rad (approximately 57°), and the average phase difference is about 0.5 rad (about 28°). The average cross-polarization conversion ratio of the original design is 0.929, while that of the +10 nm sample is 0.788, showing an average decrease of about 15.3%. Most units still maintain a relatively high cross-polarization conversion ratio, but a few units exhibit a significant decrease, with the maximum dropping from 0.971 to 0.346, a decrease of about 64%. Detailed comparison data are provided in Supplementary Table S13. The above results indicate that the dimensional error mainly affects the polarization conversion efficiency, while its impact on the target OAM mode is relatively limited. Therefore, the design has a certain tolerance for the target OAM mode, but the polarization conversion efficiency is relatively sensitive to dimensional errors; in large-area fabrication, the dimensional uniformity of the nanopillars must be strictly controlled. A dimensional control of +10 nm is within the capability of standard electron-beam lithography or deep-ultraviolet lithography.

For the sample with both length and width reduced by 10 nm, the main-ring intensity profile and the three-peak structure of the OAM spectrum are essentially consistent with the original design, with no obvious degradation. For the main-ring radius errors at five propagation planes, the errors of the original design are 15.41%, 6.41%, 3.28%, 1.27%, and 0.47%, respectively, while those of the −10 nm sample are 15.57%, 6.14%, 3.39%, 1.27%, and 0.47%, respectively. The two sets of results almost completely overlap, with a maximum difference of only about 0.16%. The OAM purity remains essentially unchanged, and the OAM spectrum of the sector-encoded plane still retains a clear three-peak structure, with a maximum variation in the total proportion of the three peaks of about 0.34%. The total transmittance changes from 0.702 to 0.738, with a variation of less than 6%. The above results indicate that the design can still maintain the target OAM mode under a dimensional deviation of ±10 nm, while the polarization conversion efficiency is relatively sensitive to dimensional errors.

To more comprehensively verify the robustness of the vector polarization characteristics, we have supplemented the Stokes-parameter distributions and polarization extinction ratio (PER) analysis under ±10 nm dimensional errors and ±10 nm wavelength drift.

**PER under ±10 nm dimensional deviations.**
We considered two cases: the nanopillar length and width are both increased by 10 nm (LW+10 nm) and both decreased by 10 nm (LW−10 nm ), and recalculated the PER within the target OAM channels. As shown in Figure S7, across the five propagation planes, the PER profiles within the target sectors and target OAM channels for both positive and negative dimensional deviations highly coincide with the unperturbed baseline profile, and the PER consistently remains at a high level (well above 10 dB). This indicates that the ±10 nm length/width dimensional deviations do not destroy the dominance of the target spin, and the orthogonal-spin leakage is still effectively suppressed.

**PER under ±10 nm wavelength drift.**
We shifted the operating wavelength from the central wavelength of 633 nm to 623 nm and 643 nm, and re-extracted the PER on the main ring. As shown in Figure S7 for 623 nm and 643 nm, the PER distributions within the target channels across the five propagation planes are highly consistent with those at the central wavelength of 633 nm. A slight decrease only occurs at the edge regions in the early propagation stage (e.g., z = 15 μm), while the spin purity in the central main-ring region remains extremely high, demonstrating the good tolerance of the device to wavelength drift.

**Stokes parameters under ±10 nm wavelength drift.**
To directly observe the influence of wavelength drift on the vector polarization state, Figure S8 shows the normalized Stokes parameters s_1_ , s_2_ , s_3_ at 623 nm across the five propagation planes. It can be seen that, within the designed linear-polarization sectors, s_3_ remains close to 0, the polarization azimuth ψ maintains a clear linear variation along the azimuthal direction, and the ellipticity ∣χ∣ also remains close to 0, confirming that the polarization state remains stable under wavelength drift.

**Substrate effect**. All FDTD simulations have already included the SiO₂ substrate. Its refractive index (about 1.45 at 633 nm) introduces a small amount of reflection at the substrate–air interface, which has been accounted for in the total transmittance T_MS_=0.702. The influence of the substrate on the phase profile and propagation characteristics of the Bessel beam is negligible, because the beam is generated in the transmission direction and the substrate is optically thick but low-loss. No additional guided modes or significant scattering were observed in the simulations. Therefore, the substrate does not compromise the design functionality.

The current design is optimized for a single wavelength of 633 nm. The performance under wavelength drifts of −10 nm and +10 nm (623 nm and 643 nm) was evaluated. For 623 nm, the maximum variation in OAM purity is about 1.4%, and the total transmittance is 0.697, with a variation of less than 1%. The main-ring radius of the Bessel beam is approximately proportional to the wavelength, with a theoretical variation of about 1.6%. At 623 nm, the errors between the measured and theoretical main-ring radii at the five propagation planes are 13.73%, 4.08%, 2.20%, 1.01%, and 1.13%, respectively. For comparison, the corresponding errors at 633 nm are 15.41%, 6.41%, 3.28%, 1.27%, and 0.47%, respectively. The difference between the left and right peak positions in the intensity profile does not exceed 0.067 μm, indicating that the radial symmetry remains well preserved.

For 643 nm, the OAM purity, main-ring intensity profile, and three-peak structure also remain stable, and the main-ring radius variation is consistent with the theoretical prediction. The errors between the measured and theoretical main-ring radii at the five propagation planes are 17.66%, 4.09%, 2.33%, 3.21%, and 1.13%, respectively. For comparison, the corresponding errors at 633 nm are 15.41%, 6.41%, 3.28%, 1.27%, and 0.47%, respectively. The difference between the left and right peak positions remains within an acceptable range, and the polarization topology distribution of the vector beam can still be clearly identified. The above results indicate that the device has a wavelength tolerance of about ±10 nm near 633 nm.

**Angular bandwidth.** The metasurface is designed for normal incidence. The incidence angle was varied from 0° to +2° and +5° for evaluation. For +2° incidence, the main-ring intensity profile, OAM spectrum, and polarization topology distribution remain essentially stable, and the center shift and intensity deformation are within an acceptable range, indicating that the device can operate normally within ±2°. For +5° incidence, the first two propagation planes exhibit an obvious center shift and intensity elliptization; from the third propagation plane onward, the center shift further increases and the intensity distribution is severely deformed, indicating significant degradation of the Bessel beam profile. These results indicate that the device is only suitable for small-angle incidence, and the optimal operating condition is normal incidence; in practical applications, the incidence angle should be kept within ±2° as much as possible.

**Impact of high *L* values on aperture size and fabrication challenges.**For high-order Bessel beams, the main-ring radius is approximately

For *L*=220,=633 nm and NA=0.5, we obtain µm, and the main ring falls entirely within the designed aperture of 214 µm × 214 µm . This aperture is sufficient for all the L values used (up to 220). However, a large aperture requires a large number of nanopillars. With a period of 420 nm, the 214 μm aperture contains approximately 511 × 511nanopillars, which is within the capability of standard electron-beam lithography.

**Supplementary Table S11.** **compares the main-ring radius errors between the +10 nm sample and the original design at five propagation planes.**

| z(μm) | 15 | | 42 | | 69 | | 100 | | 125 | |
| --- | --- | --- | --- | --- | --- | --- | --- | --- | --- | --- |
| Side | *-x* | *+x* | *-x* | *+x* | *-x* | *+x* | *-x* | *+x* | *-x* | *+x* |
| Measured center *x* (μm) | 8.957 | 8.991 | 11.526 | 11.526 | 13.561 | 13.561 | 12.16 | 12.16 | 10.559 | 10.559 |
| Center difference (good radial symmetry) | 0.034 | | 0 | | 0 | | 0 | | 0 | |
| Net topological charge *L*: phase order | 60 | | 70 | | 80 | | 70 | | 60 | |
| Theoretical derivative zero (asymptotic) | 63.1656174 | | 73.33252965 | | 83.48421279 | | 73.33252965 | | 63.1656174 | |
| Theoretical derivative zero (exact MATLAB value) | 63.183304 | | 73.349409 | | 83.500416 | | 73.349409 | | 63.183304 | |
| Theoretical radius (exact value × 633/2πNA), NA = 0.6 | 10.60901177 | | 12.31598688 | | 14.02042691 | | 12.31598688 | | 10.60901177 | |
| Measured radius (μm): first increases then decreases | 8.974 | | 11.526 | | 13.561 | | 12.16 | | 10.559 | |
| Absolute deviation between measured radius and theoretical value | 1.635011767 | | 0.789986881 | | 0.459426913 | | 0.155986881 | | 0.050011767 | |
| Relative error | **15.41%** | | **6.41%** | | **3.28%** | | **1.27%** | | **0.47%** | |

| z(μm) | 15 | | 42 | | 69 | | 100 | | 125 | |
| --- | --- | --- | --- | --- | --- | --- | --- | --- | --- | --- |
| Side | *-x* | *+x* | *-x* | *+x* | *-x* | *+x* | *-x* | *+x* | *-x* | *+x* |
| Measured center *x* (μm) | 8.957 | 9.024 | 11.593 | 11.493 | 13.595 | 13.528 | 12.16 | 12.193 | 10.559 | 10.559 |
| Center difference (good radial symmetry) | 0.067 | | 0.1 | | 0.067 | | 0.033 | | 0 | |
| Net topological charge *L*: phase order | 60 | | 70 | | 80 | | 70 | | 60 | |
| Theoretical derivative zero (asymptotic) | 63.1656174 | | 73.33252965 | | 83.48421279 | | 73.33252965 | | 63.1656174 | |
| Theoretical derivative zero (exact MATLAB value) | 63.183304 | | 73.349409 | | 83.500416 | | 73.349409 | | 63.183304 | |
| Theoretical radius (exact value × 633/2πNA), NA = 0.6 | 10.60901177 | | 12.31598688 | | 14.02042691 | | 12.31598688 | | 10.60901177 | |
| Measured radius (μm): first increases then decreases | 8.9905 | | 11.543 | | 13.5615 | | 12.1765 | | 10.559 | |
| Absolute deviation between measured radius and theoretical value | 1.618511767 | | 0.772986881 | | 0.458926913 | | 0.139486881 | | 0.050011767 | |
| Relative error | **15.26%** | | **6.28%** | | **3.27%** | | **1.13%** | | **0.47%** | |

**Supplementary Table S12. Comparison of the three OAM peaks in the OAM spectra between the original design and the +10 nm sample**

| Propagation distance z | LCP three-peak structure | Sum of three peaks | RCP three-peak structure | Sum of three peaks | Net OAM L |
| --- | --- | --- | --- | --- | --- |
| 15 μm | 59(0.259) 60(0.269) 61(0.298) | 0.826 | 59(0.261) 60(0.450) 61(0.193) | 0.904 | 60 |
| 15 μm（pillar+10nm） | 61(0.302) 60(0.267) 59(0.252) | 0.821 | 60(0.453) 59(0.261) 61(0.194) | 0.908 | 60 |
| 42 μm | 69(0.282) 70(0.407) 71(0.225) | 0.914 | 69(0.329) 70(0.315) 71(0.194) | 0.838 | 70 |
| 42 μm(pillar+10nm) | 70(0.401) 69(0.273) 71(0.235) | 0.909 | 69(0.326) 70(0.318) 71(0.190) | 0.834 | 70 |
| 69 μm | 79(0.892) | 0.892 | 81(0.868) | 0.868 | 80 |
| 69 um(pillar+10nm) | 79(0.891) | 0.891 | 81(0.870) | 0.87 | 80 |
| 100 μm | 69(0.247)  70(0.308)  71(0.300) | 0.855 | 69(0.295) 70(0.393) 71(0.208) | 0.896 | 70 |
| 100 um  (pillar+10nm) | 70(0.308) 71(0.305) 69(0.234) | 0.847 | 70(0.399) 69(0.286) 71(0.205) | 0.89 | 70 |
| 125 μm | 59(0.283) 60(0.385) 61(0.245) | 0.913 | 59(0.332) 60(0.351) 61(0.190) | 0.873 | 60 |
| 125 μm  (pillar+10nm) | 60(0.387) 59(0.272) 61(0.249) | 0.908 | 60(0.354) 59(0.327) 61(0.187) | 0.868 | 60 |

**Supplementary Table S13. Phase difference and cross-polarization efficiency of the +10 nm sample**

| xphase | xT | yphase | yT | Phase difference | Cross-polarization efficiency |
| --- | --- | --- | --- | --- | --- |
| 0.405342 | 0.756463 | 4.4512152 | 0.952869 | 4.0458732 | 0.691532147 |
| 1.23245 | 0.976998 | 4.6129952 | 0.981182 | 3.3805452 | 0.965180276 |
| 1.64926 | 0.990092 | 5.0022752 | 0.997775 | 3.3530152 | 0.982867731 |
| 2.34815 | 0.99926 | 5.6059042 | 0.999175 | 3.2577542 | 0.995850552 |
| 2.46044 | 0.998481 | 6.2635633 | 0.807351 | 3.8031233 | 0.807682218 |
| 3.5694952 | 0.967211 | 5.2025552 | 0.334919 | 1.63306 | 0.345788255 |
| 4.4512352 | 0.952888 | 0.40535 | 0.756464 | 4.0458852 | 0.691536208 |
| 4.6490852 | 0.980756 | 1.32617 | 0.979174 | 3.3229152 | 0.971932255 |
| 5.3843112 | 0.959411 | 1.52502 | 0.985695 | 3.8592912 | 0.852598935 |
| 5.5934982 | 0.99145 | 2.20595 | 0.999564 | 3.3875482 | 0.980527162 |
| 5.0896752 | 0.351272 | 2.64178 | 0.995589 | 2.4478952 | 0.59561129 |
| 5.6083342 | 0.646251 | 3.6247952 | 0.977863 | 1.983539 | 0.568895946 |

Figure S3 Phase responses of the original design and the +10 nm sample. The *x*-phase and *y*-phase of the original design are represented by purple squares (phix) and blue upward triangles (phiy), respectively; the *x*-phase and *y*-phase of the +10 nm sample are represented by red circles (xphase) and green downward triangles (yphase), respectively. The horizontal axis denotes the nanopillar index, and the vertical axis denotes the phase (in rad).

**Supplementary Note S13. OAM Spectrum Analysis**

**S13.1 Principle of angular Fourier decomposition**

To quantitatively characterize the OAM composition of the transmitted field, the complex amplitude *E*(*r*,*θ*) within the main-ring region is decomposed into spiral harmonics by angular Fourier transform:

where the Fourier coefficient is given by

The quantity represents the power density of the OAM channel with topological charge *l* at radius *r*. After radial integration, the total power of this OAM channel is

The normalized OAM power spectrum is then defined as

**S7.2 Sector-wise phase gradient analysis**

To further verify that the local phase gradient within each sector exactly equals the designed value, we extract the actual topological charge of each half-sector by least-squares linear fitting. The detailed procedure is as follows.

Angular sampling is performed along the mid-radius of the main ring, A total of 720 data points are collected along the azimuthal direction, yielding the unwrapped phase *ϕ*(*θ*). For each half-sector, the phase is fitted by

where the fitted slope *l* directly gives the actual topological charge of that sector.

As a complementary verification, the unwrapped phase was numerically differentiated to obtain the instantaneous phase gradient, d*ϕ*/d*θ*. The average value of this instantaneous gradient was then compared with the fitted slope obtained from the polyfit of *ϕ*(*θ*). The instantaneous slope curve fluctuates slightly around the fitted slope, and its mean value agrees well with the fitted value. This confirms the local uniformity of the phase gradient.

**Supplementary Note S14.** **Asymptotic Bessel zeros and two definitions of the main-ring radius**

The field amplitude of a Bessel beam is

**Definition 1: field-zero radius.**

In the literature, the main-ring radius is often defined as the radial position of the first zero of the field amplitude:

where is the first positive zero of .This position corresponds to an intensity minimum.

**Definition 2: intensity-peak radius.**
In simulations and experiments, the radius is usually extracted from the intensity maximum.

where  is the first positive zero of .This position corresponds to the intensity maximum.

The high-order asymptotic expansions are

$$j_{l,1}\sim l+a_{1}(\frac{l}{2})^{1/3}+\cdots,a_{1}\approx2.33811$$

$$j_{l,1}^{'}\sim l+a_{1}^{'}(\frac{l}{2})^{1/3}+\cdots,a_{1}^{'}\approx1.01879$$

In this work, both definitions are provided for the theoretical radius. The measured radius is extracted from the intensity maximum and is therefore compared with .

**Supplementary Note S15. Exploratory study of the dual-ring design**

This note presents an exploratory study of the dual-ring design. This case is used to examine possible radius errors and structural constraints in multi-channel extension, and is not presented as direct evidence that the proposed scheme has achieved multi-channel scalability. In the dual-ring design, the relative outer-ring radius error decreases from 30.9% to 8.9% over the investigated propagation range, which is significantly larger than the inner-ring error (from 13.8% to 0.8%). This difference indicates that the outer ring is more sensitive to phase discretization and effective NA variation.

**S15.1 Dual-ring layout and phase distributions**

The dual-ring design employs two concentric rings, each independently encoding its target *L* and *P.* The inner ring uses *NA*_1_=0.6 with an *L* sequence of 100, 105, 110, 105, 100; the outer ring uses *NA*_2_=0.5 with an *L* sequence of 200, 205, 210, 215, 220. Both rings adopt *M*=2 azimuthal sector encoding, with polarization orders $P=+1$ and $P=-1$, respectively. Figure S4 (a) shows the phase profiles of the inner and outer rings; Figure S4(b) shows the radial-to-longitudinal mapping established by the axicon phase. To ensure that both the inner and outer concentric main rings remain within the non-diffracting range over the same observation interval $Z_{obs}$, different radial sampling granularities are adopted for the inner and outer ring widths in the mapping design.

Figure S4 (a) Designed phase distributions and for $MS_{2}$. The left colorbar indicates discrete $m$ and $n$ values. The $L$ sequences correspond distinct longitudinal radius evolution trends: monotonic ($L_{4}-L_{8}$). Column 1 shows the topological charge ($m$ or $n$) distribution, column 2 the azimuthal angle , and column 3 the $NA$-dependent axicon phase; the vortex phase is or .(b) Linear $\rho-z$ mapping imposed by the axicon phase. $NA_{1}$ and $NA_{2}$ correspond to different mapping slopes for the inner and outer rings of beam for $MS_{2}$.

**S15.2 Intensity distributions under different linear polarizations**

Figure S5 shows the intensity distributions of the dual-ring beam under different linear polarization directions. On the target propagation planes, both the inner and outer rings exhibit lobe rotation consistent with the designed polarization orders; the rotation directions of the two rings are determined by the signs of their respective *P*.

Figure S5 Transverse intensity distributions for five linear polarization directions at five propagation distances. The polarization directions are indicated by blue arrows. The inner and outer ring sectors are independently assigned polarization orders *P*. For each plane, the five polarization-resolved intensity images are normalized to the maximum intensity among the five polarization states, and all subfigures share the same color bar (0 → 1) representing relative intensity. The coordinate range for all subfigures is ±80 µm in both *x* and *y.*

**S15.3 Inner- and outer-ring radius errors versus propagation distance**

Figure S6 shows the inner- and outer-ring radius errors as functions of propagation distance. The inner-ring error decreases from 13.84% to 0.82%, while the outer-ring error decreases from 30.94% to 8.85%. The inner-ring error converges relatively quickly with propagation distance, whereas the outer-ring error remains relatively large over the investigated range, indicating that the outer ring is more sensitive to phase discretization and effective NA variation.

Figure S6 Left and right subpanels show, for the inner ring and the outer ring respectively, the theoretical main ring radius (green bars), the measured radius (light orange bars), and the relative radius error (blue line with markers) as functions of the propagation distance.

**S15.4 Dependence of the outer-ring error on the outer-ring NA**

To quantitatively analyze the origin of the outer-ring error, three dual-ring metasurfaces A, B, and C were designed with identical inner-ring parameters but different outer-ring NAs.

**Supplementary Table S14 Inner- and outer-ring parameters and radius errors of the three dual-ring metasurfaces (A, B, and C).**

| Parameter | Metasurface A | Metasurface B | Metasurface C |
| --- | --- | --- | --- |
| Inner-ring NA₁ | 0.6 | 0.6 | 0.6 |
| Inner-ring L sequence | 100/105/110/105/100 | 100/105/110/105/100 | 100/105/110/105/100 |
| Inner-ring error (five propagation planes) | 13.84%, 7.26%, 5.79%, 3.61%, 0.82% | 13.84%, 7.99%, 4.05%, 2.51%, 0.82% | 14.22%, 7.62%, 5.79%, 3.60%, 0.43% |
| Outer-ring NA₂ | 0.5 | 0.4 | 0.6 |
| Outer-ring L sequence | 200/205/210/215/220 | 200/205/210/215/220 | 200/205/210/215/220 |
| Outer-ring error (five propagation planes) | 30.94%, 20.13%, 13.68%, 9.93%, 8.85% | 38.28%, 29.66%, 20.69%, 16.86%, 15.89% | 20.62%, 12.11%, 4.92%, 4.21%, 3.37% |

Metasurface A: outer ring *NA*_2_=0.5, outer-ring errors of 30.94%, 20.13%, 13.68%, 9.93%, and 8.85%;Metasurface B: outer ring *NA*_2_=0.4, outer-ring errors of 38.28%, 29.66%, 20.69%, 16.86%, and 15.89%; Metasurface C: outer ring *NA*_2_=0.6, outer-ring errors of 20.62%, 12.11%, 4.92%, 4.21%, and 3.37%.

The results show that the outer-ring error increases significantly as the outer-ring NA decreases. The outer-ring error of metasurface B (38%→15%) is clearly larger than those of A (30.9%→8.9%) and C (20.6%→3.4%). In terms of the ratio of the ring radius to the aperture radius: for *NA*_2_=0.6, the theoretical radius is about 34–37 μm, with a ratio of 0.31–0.34; for *NA*_2_=0.5, about 41–45 μm, with a ratio of 0.38–0.42; for *NA*_2_=0.4, about 51–56 μm, with a ratio of 0.48–0.52. The metasurface aperture has a diameter of 214 μm and a radius of 107 μm. In all three cases, the ratio of the outer-ring radius to the aperture radius is well below the 0.8 threshold commonly regarded as significant for truncation effects. Therefore, finite-aperture truncation is not the main origin of the outer-ring error. The outer-ring error mainly originates from the NA-dependent phase-gradient error: the smaller the NA, the smaller the axicon phase gradient, and the larger the proportion of the same phase discretization and sector-boundary compensation errors relative to the phase signal, leading to a larger deviation of the main-ring position.

**S15.5 Control results of the outer-ring-only design**

To further separate the contributions of dual-ring interference and the outer-ring intrinsic error, a control metasurface D retaining only the outer-ring phase and removing the inner-ring phase was designed. For *NA*_2_=0.5, the outer-ring errors of the outer-ring-only design are 30.45%, 19.49%, 14.75%, 9.33%, and 9.15%, which are almost identical to the outer-ring errors of dual-ring metasurface A (30.94%, 20.13%, 13.68%, 9.93%, and 8.85%), with a difference of less than about 0.5 percentage points. This indicates that the presence of the inner ring contributes almost no additional error to the outer ring, and that the outer-ring error mainly originates from the outer ring itself, namely phase discretization and NA-dependent phase-gradient error, rather than dual-ring interference.

**S15.6 Effect of the metasurface aperture on the outer-ring error**

To examine the effect of the metasurface aperture on the outer-ring error, two outer-ring-only designs with apertures of 214 μm and 176 μm were compared. Both designs use the same outer-ring parameters: NA= 0.5, *L* sequence 200, 205, 210, 215, 220, and wavelength 633 nm. The results show that, for *L* = 200–215, the outer-ring errors under the two apertures are almost identical, with a maximum difference of less than about 0.2 percentage points. At *L* = 220, the outer-ring error decreases from 9.15% for the 511 aperture to 4.44% for the 421 aperture. This indicates that, for *L* = 200–215, the aperture has little effect on the outer-ring error, and the outer-ring error mainly originates from the outer ring’s own phase discretization and phase-gradient error. At *L* = 220, where the order is higher, the aperture starts to play a non-negligible role, but the outer-ring error is still overall dominated by the outer ring’s own characteristics.

**S15.7 Limitations and future work**

The available results show that the outer-ring error in the dual-ring design is relatively large, and that the two rings sharing the same aperture and NA further compresses the available design space, since their radial separation relies entirely on the difference in *L.*Therefore, the dual-ring design is not presented in this note as direct evidence for multi-channel scalability. Inter-ring crosstalk, dual-ring OAM spectra, and the constraints among the number of channels, NA, aperture, and maximum OAM order will be quantified in future work through multiple metasurface groups.

Figure S7. Simulated polarization extinction ratios (PER) in the spin–OAM joint domain under ±10 nm dimensional deviations and ±10 nm wavelength drift.This figure shows the transverse PER distributions (in dB) at five specific propagation distances (including z=15,42,69,95,125 μm). Each column corresponds to the central wavelength of 633 nm (left column) and the shifted wavelengths of 623 nm (middle column) and 643 nm (right column). The bottom two rows show the results when the nanopillar length and width are both increased by 10 nm (LW+10 nm) and both decreased by 10 nm (LW−10 nm), respectively. Curves of different colors in each subplot represent different sectors and target OAM channels (e.g., l=59,61 or 69,71, etc., see legends for specific correspondence). The results show that the PER distribution patterns within the target channels are not destroyed by the LW±10 nm dimensional deviations and ±10 nm wavelength drift, and the orthogonal-spin leakage is effectively suppressed.

Figure S8 Simulated vector polarization states at 623 nm across five propagation planes under wavelength drift. The rows (from top to bottom) show the normalized Stokes parameters s_1_, s_2_, s_3_, the ellipticity ∣χ∣, and the polarization azimuth ψ, respectively. The columns (z_1_ to z_5_) correspond to five specific propagation distances. The color bars on the right indicate the numerical values of the respective parameters. Within the designed linear-polarization sectors, s_3_ and the ellipticity ∣χ∣remain close to 0, while the azimuth ψ exhibits a clear linear variation along the azimuthal direction. This confirms that the vector polarization state remains stable against wavelength drift.
